# Vascular smooth muscle–specific YAP/TAZ deletion triggers aneurysm development in mouse aorta

**DOI:** 10.1172/jci.insight.170845

**Published:** 2023-09-08

**Authors:** Marycarmen Arévalo Martínez, Olivia Ritsvall, Joakim Armstrong Bastrup, Selvi Celik, Gabriel Jakobsson, Fatima Daoud, Christopher Winqvist, Anders Aspberg, Catarina Rippe, Lars Maegdefessel, Alexandru Schiopu, Thomas A. Jepps, Johan Holmberg, Karl Swärd, Sebastian Albinsson

**Affiliations:** 1 Vascular Physiology Environment, Department of Experimental Medical Science, Lund University, Lund, Sweden.; 2 Vascular Biology Group, Department of Biomedical Sciences, University of Copenhagen, Copenhagen, Denmark.; 3 Molecular Cardiology, Department of Clinical Sciences, Lund University, Lund, Sweden.; 4 Department of Translational Medicine, Lund University, Malmö, Sweden.; 5 Rheumatology and Molecular Skeletal Biology, Department of Clinical Sciences Lund, Lund University, Lund, Sweden.; 6 Department of Medicine, Karolinska Institute, Stockholm, Sweden, and; 7 Department of Vascular and Endovascular Surgery, Klinikum rechts der Isar - Technical University Munich (TUM), Munich, Germany.; 8 Department of Internal Medicine, Skåne University Hospital Lund, Lund, Sweden, and; 9 Nicolae Simionescu Institute of Cellular Biology and Pathology, Bucharest, Romania.

**Keywords:** Cell Biology, Vascular Biology, Cardiovascular disease, Hypertension, Vasculitis

## Abstract

Inadequate adaption to mechanical forces, including blood pressure, contributes to development of arterial aneurysms. Recent studies have pointed to a mechanoprotective role of YAP and TAZ in vascular smooth muscle cells (SMCs). Here, we identified reduced expression of *YAP1* in human aortic aneurysms. Vascular SMC–specific knockouts (KOs) of YAP/TAZ were thus generated using the integrin α8–*Cre* (*Itga8*-*Cre*) mouse model (i8-YT-KO). i8-YT-KO mice spontaneously developed aneurysms in the abdominal aorta within 2 weeks of KO induction and in smaller arteries at later times. The vascular specificity of *Itga8-Cre* circumvented gastrointestinal effects. Aortic aneurysms were characterized by elastin disarray, SMC apoptosis, and accumulation of proteoglycans and immune cell populations. RNA sequencing, proteomics, and myography demonstrated decreased contractile differentiation of SMCs and impaired vascular contractility. This associated with partial loss of myocardin expression, reduced blood pressure, and edema. Mediators in the inflammatory cGAS/STING pathway were increased. A sizeable increase in SOX9, along with several direct target genes, including aggrecan (*Acan*), contributed to proteoglycan accumulation. This was the earliest detectable change, occurring 3 days after KO induction and before the proinflammatory transition. In conclusion, *Itga8-Cre* deletion of YAP and TAZ represents a rapid and spontaneous aneurysm model that recapitulates features of human abdominal aortic aneurysms.

## Graphical abstract

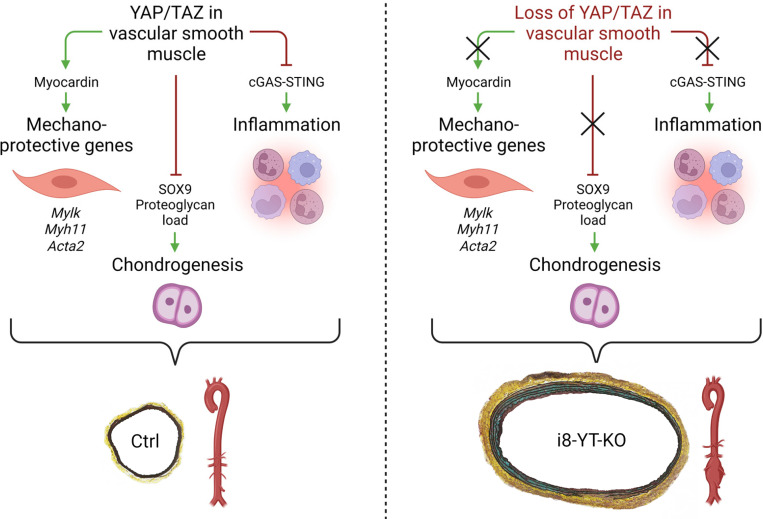

## Introduction

Hypertension is one of the major contributors to the global burden of disease and disability (1), acting by accelerating numerous cardiovascular disorders, including atherosclerosis and aneurysm formation (2). The cardiovascular system adapts in multiple ways to an elevated blood pressure (2), and recent work has highlighted a role for the mechanoresponsive proteins YAP (gene symbol, *Yap1*) and TAZ (gene symbol, *Wwtr1*) in this adaptation (3–6). YAP and TAZ are transcriptional coactivators under negative control of a kinase cascade called the Hippo pathway (7–9). They are highly expressed in the arterial wall (2), and upon mechanical challenge, YAP and TAZ move to the nucleus (4, 10), where they interact with TEA-domain transcription factors to cause changes in gene expression (11).

In recent work, we (3, 12) and others (5, 13) created cell type–specific and tamoxifen-inducible knockouts (KOs) of YAP and TAZ using the *Myh11-CreER^T2^* mouse (14). *Myh11-CreER^T2^* effectively recombines floxed alleles in vascular and visceral smooth muscle cells (SMCs) (15, 16). As is often the case with inducible disruption of genes essential for SMC function (17, 18), YAP/TAZ deletion led to severe intestinal obstruction (12, 13). Obstruction required deletion of all 4 alleles of *Yap1* and *Wwtr1* (12), indicating that the coactivators can substitute for each other. The intestinal phenotype forced us to euthanize mice at 9 to 11 days (12) and limited detection and characterization of vascular phenotypes. A window of opportunity was nonetheless present, allowing for detection of reduced contractility and differentiation of SMCs (3, 13). Moreover, when subjected to angiotensin II–induced hypertension, we found that the *Myh11-CreER^T2^*–driven YAP/TAZ-KO mice developed aneurysm-like lesions at staggering speed (3). This suggests that YAP/TAZ-dependent vascular adaptation to hypertension is critical for vascular patency.

Another study, using a different induction protocol, found spontaneous aortic aneurysms in the *Myh11-CreER^T2^*–YAP/TAZ-KO model and attributed this to cyclic guanosine monophosphate–adenosine monophosphate synthase (cGAS)/stimulator of interferon genes (STING) activation (5). cGAS/STING is an arm of innate immunity that responds to double-stranded DNA in the cytoplasm (19, 20) and that activates tank-binding kinase 1 (TBK1) to effect changes in NF-κB–driven proinflammatory transcription (21). cGAS/STING activation is a feature of human aneurysmal disease (22). This is also true for mucoid extracellular matrix accumulation, apoptosis, and infiltration of immune cells in the aortic media (23), but the role of YAP and TAZ for the latter aneurysm manifestations remains to be determined.

The *Myh11-CreER^T2^* mouse (14), while being very useful, causes a severe gastrointestinal disease, which confounds and limits interpretation of vascular phenotypes. To overcome this issue, the integrin α8 (*Itga8*) promoter was used for transgenic Cre expression (17). *Itga8* is preferentially expressed in vascular over visceral SMCs (24), and the *Itga8-Cre* model evaded a severe visceral myopathy when used to delete serum response factor (SRF) (17). In the present work, we capitalized on the advantages of the *Itga8-Cre* mouse for KO of YAP and TAZ in vascular SMCs. A rapid and severe aneurysmal phenotype was discovered, and we used transcriptomic and proteomic analyses to pinpoint the molecular mechanisms underlying aortic disease. *Itga8-Cre*–driven deletion of YAP and TAZ caused loss of arterial contractility, pronounced SOX9 induction, along with accumulation of the proteoglycan aggrecan. We also detected proinflammatory TBK1 activation with infiltration of myeloid and lymphoid immune cells, including monocytes and macrophages. This mouse model therefore recapitulates features of aortic aneurysms in humans. Indeed, our results indicate that development of human abdominal aortic aneurysm (AAA) associates with depletion of YAP.

## Results

### i8-YT-KO mice develop aneurysms within weeks of KO induction.

**Figure 1:**
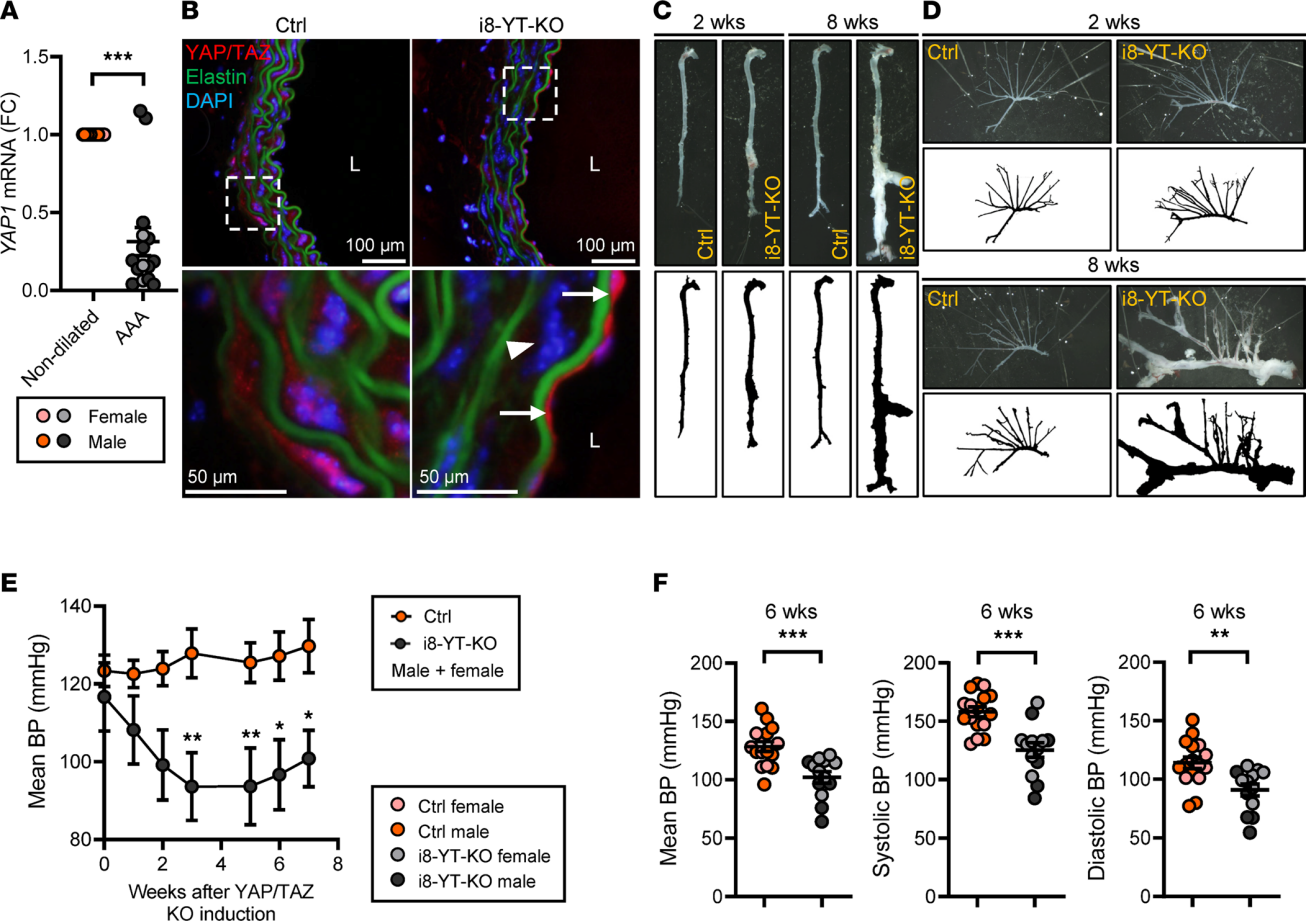
i8-YT-KO mice develop severe vascular lesions. (**A**) *YAP1* levels were determined by RT-qPCR in human control (nondilated) aortae and in abdominal aortic aneurysms (AAAs). To examine the role of YAP/TAZ in aneurysm development, the i8-YT-KO mouse was generated. (**B**) Immunofluorescence images of YAP/TAZ (red) in the aorta at 2 weeks after tamoxifen injection to induce knockout. Reduced YAP/TAZ labeling of smooth muscle cells (arrowhead), but not of endothelial cells (arrows), is apparent in i8-YT-KO aorta. Elastin, which is autofluorescent, is shown in green and DAPI nuclear stain in blue. (**C**) Photomicrographs (top) and black-and-white (bottom) images of dissected aortae from control and i8-YT-KO mice at 2 and 8 weeks following YAP/TAZ deletion. (**D**) The mesenteric arterial tree was dissected from 2- and 8-week mice. (**E**) Mean arterial blood pressure (BP) over time, starting before tamoxifen injections, is shown (*n ≥* 6). (**F**) Mean, systolic, and diastolic blood pressures at 6 weeks from a larger cohort of mice (including mice from panel **E**) are represented as individual animals (*n ≥* 13). L, lumen. *P < 0.05; **P < 0.01; ***P < 0.001 by Mann-Whitney test (**A**), 2-way ANOVA with Bonferroni’s post hoc test (**E**), or 2-tailed Student’s *t* test (**F**).

To determine whether YAP is dysregulated in human AAAs, paired vascular tissue samples from dilated and nondilated abdominal aortae were analyzed by RT-qPCR. The *YAP1* mRNA level was reduced in dilated samples, suggesting that loss of YAP in the vascular wall associates with AAAs in humans (Figure 1A and Supplemental Table 1; supplemental material available online with this article; https://doi.org/10.1172/jci.insight.170845DS1). In order to understand whether this pathway protects from aneurysm development, 6- to 8-week-old *Yap1^fl/fl^* × *Wwtr1^fl/fl^* mice with 1 allele of the *Itga8-CreER^T2^* transgene (17) were injected intraperitoneally with tamoxifen for inducible KO of YAP and TAZ in vascular smooth muscle (i8-YT-KO) (Supplemental Figure 1, A and B). Cre-deficient *Yap1^fl/fl^* × *Wwtr1^fl/fl^* mice injected with tamoxifen were used as controls (Ctrls). Immunofluorescent staining using an antibody that detects both YAP and TAZ revealed red staining of SMCs between the elastic lamellae in the aortic media of Ctrl mice and of endothelial cells facing the vascular lumen (Figure 1B, left column). SMC staining was lost in i8-YT-KO mice 2 weeks after tamoxifen, but endothelial staining remained (Figure 1B, right column). This confirms the efficiency and specificity of the KO strategy. Whole mounts of the aorta 2 weeks after tamoxifen demonstrated irregular widening of aortic profiles and aortic aneurysms, primarily at the pararenal level of the abdominal aorta (Figure 1C and Supplemental Figure 2, A and B). At 8 weeks, most of the aorta was affected and clear aneurysms were observed in 100% of the 51 i8-YT-KO aortae that were dissected at this time point (Figure 1C and Supplemental Figure 2A). No aneurysms were observed in the 55 Ctrl mice dissected at 8 weeks. Aneurysms were detectable in additional vascular beds of i8-YT-KO mice, such as in the superior mesenteric artery (Figure 1D). Importantly, no gross intestinal phenotype was detected (Supplemental Figure 2C). Out of 224 mice used in the study, 5 died prior to the planned endpoint from unknown causes. Four of these mice were Cre positive and 1 was Cre negative. It is possible that the 4 Cre-positive mice died from ruptured aneurysms, but this was not confirmed.

**Figure 2:**
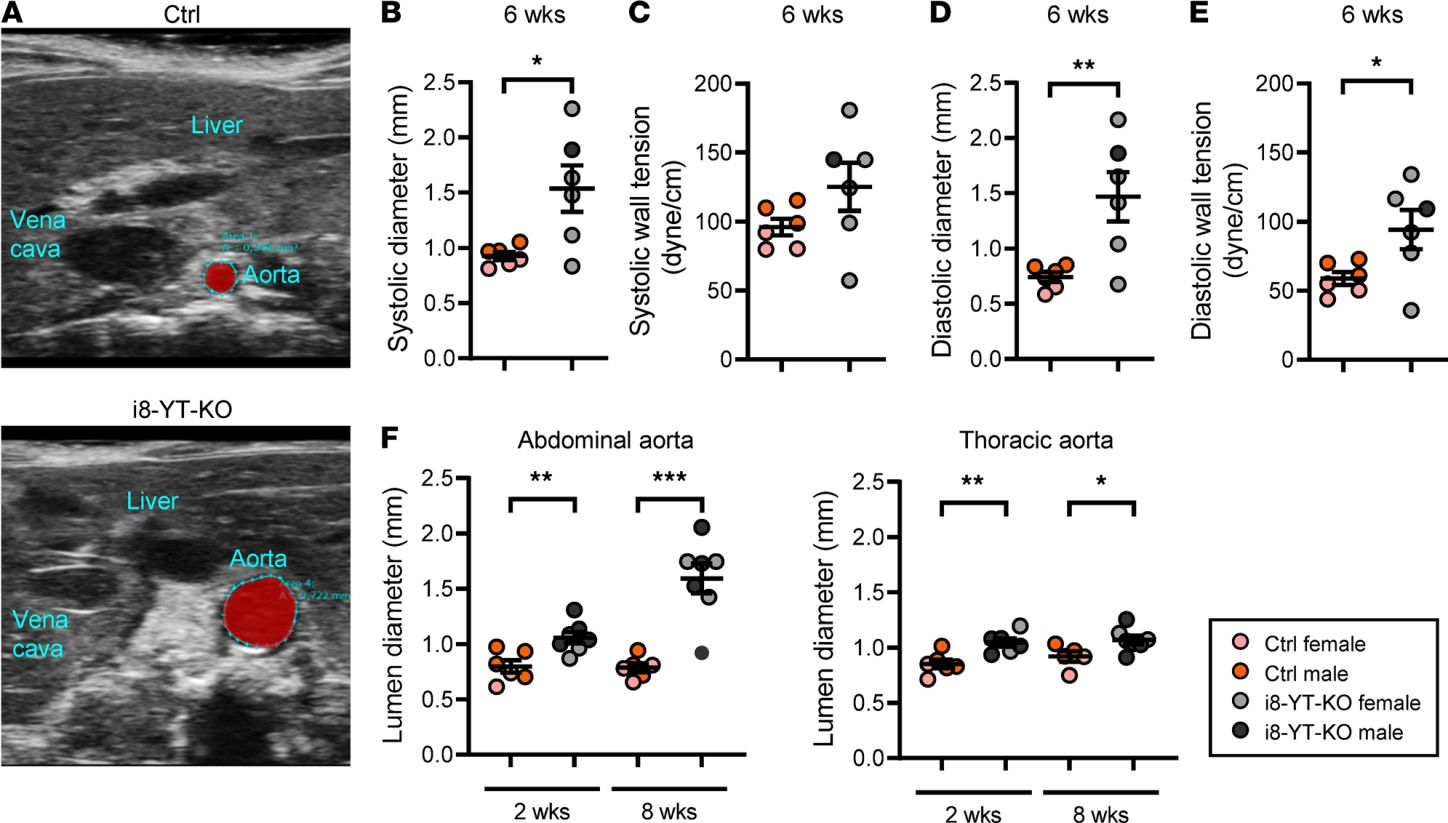
i8-YT-KO mice develop aortic aneurysms. (**A**) Echocardiography was used to determine aortic diameter in anesthetized mice. (**B**–**E**) The systolic and diastolic diameter of the abdominal aorta and systolic and diastolic wall tension at 6 weeks are shown (*n* = 6). (**F**) The aortic lumen diameter of abdominal (*n ≥* 6) and thoracic (*n ≥* 5) aortae in perfusion-fixed aortic specimens at 2 and 8 weeks. *P < 0.05; **P < 0.01; ***P < 0.001 by 2-tailed Student’s *t* test (**B**–**F**).

Blood pressure measurements using tail cuffs showed that mean arterial blood pressure was significantly reduced in male mice at 2 weeks after tamoxifen (Supplemental Figure 3) and in both males and females from 3 weeks and onwards (Figure 1E and Supplemental Figure 3). Both systolic and diastolic blood pressures were affected in another cohort (Figure 1F). Ultrasound imaging in anesthetized mice (Figure 2, A–E, and Supplemental Table 2) revealed an increased systolic aortic diameter at both 2 and 6 weeks after tamoxifen. The diastolic diameter of thoracic and abdominal aortae was significantly increased at 6 weeks, but not at the 2-week time point. Note that the measurements were performed at specific anatomical locations and do not necessarily represent the maximal diameter of the aorta. Aortic wall tension, calculated using blood pressure and diameter in individual mice (Figure 2, C and E), was increased in diastole. After perfusion-fixation, histological analysis showed an increase in the aortic lumen diameter. This was most evident in the abdominal aorta, where time-dependent progression was observed (Figure 2F). The i8-YT-KO model therefore represents a spontaneous aneurysm model that initially affects the abdominal aorta and that subsequently progresses to other arterial territories. This model is unique in that it evades a serious visceral myopathy seen in previous SMC-specific and inducible KO models for YAP and TAZ (12, 13).

### Elastin disarray, neointima formation, and cell death in aneurysms from i8-YT-KO mice.

**Figure 3:**
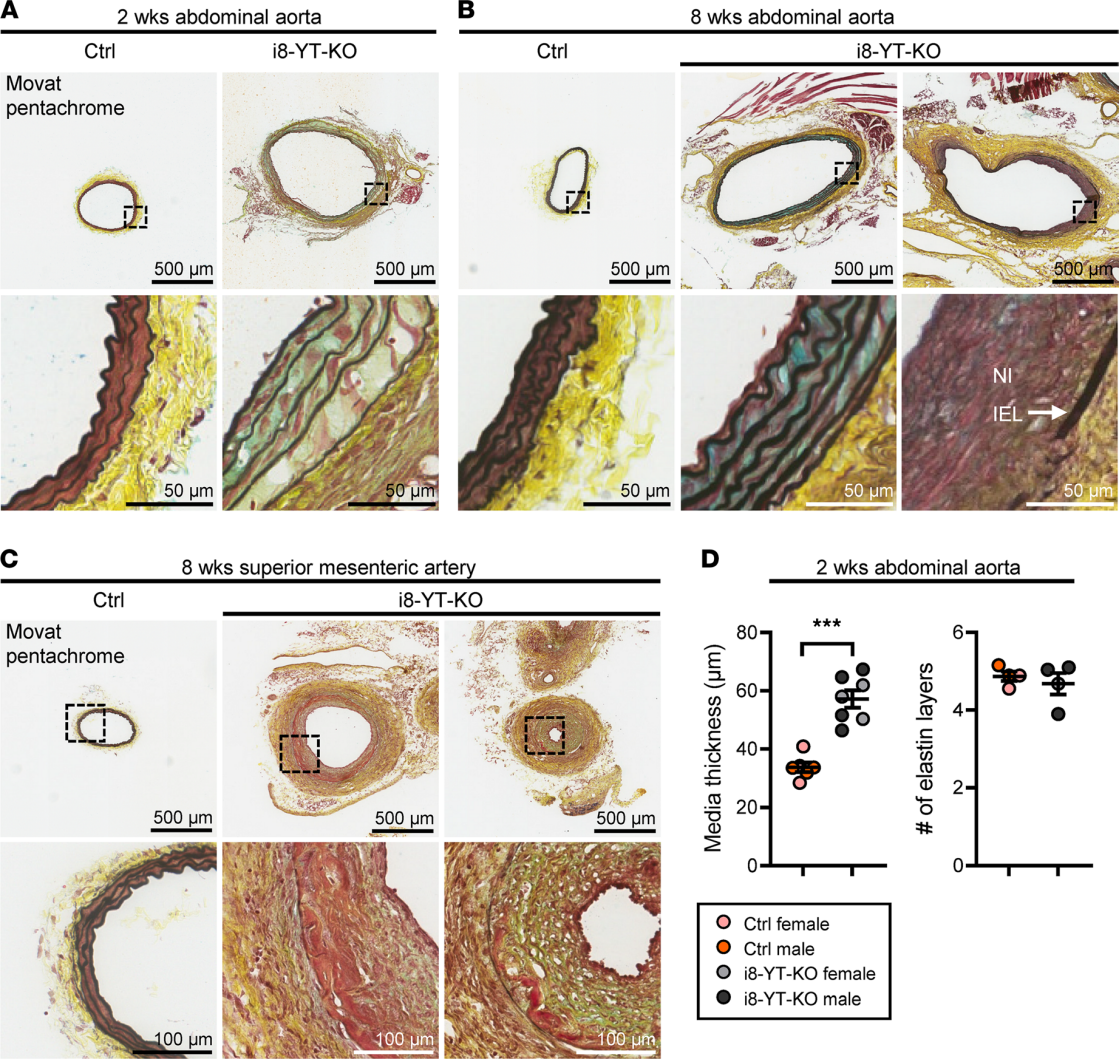
Abdominal aortic aneurysms show disorganized elastin and neointima and adventitial expansion. (**A** and **B**) Sections of the abdominal aorta at 2 and 8 weeks following the first tamoxifen injection. Movat pentachrome, which stains elastin black, collagen yellow, proteoglycans and mucins blue, and smooth muscle red, was used for staining transversally cut cryosections. Low and high magnifications are shown, and the latter are highlighted using boxes in the top row. In controls (Ctrl), medial cells in red were tightly packed between layers of elastin, and the adventitia was bright yellow. In knockout aorta (i8-YT-KO), the lumen circumference was enlarged, elastic lamellae more widely spaced, and there were fewer red medial cells, while blue staining was increased. The adventitia was more cellular. Advanced lesions in i8-YT-KO aortae were characterized by greater enlargement of the lumen diameter, advanced neointima formation (NI) inside the internal elastic lamina (IEL), and further elastin disarray with breaks in the continuity of individual lamellae. (**C**) Movat pentachrome–stained sections of lesions in the superior mesenteric artery. Neointima formation often encroached on the lumen, and black lamellae were sometimes difficult to identify. (**D**) Quantification of media thickness and the average number of lamellae in abdominal aorta at 2 weeks (*n ≥* 4). ***P < 0.001 by 2-tailed Student’s *t* test (**D**).

**Figure 4:**
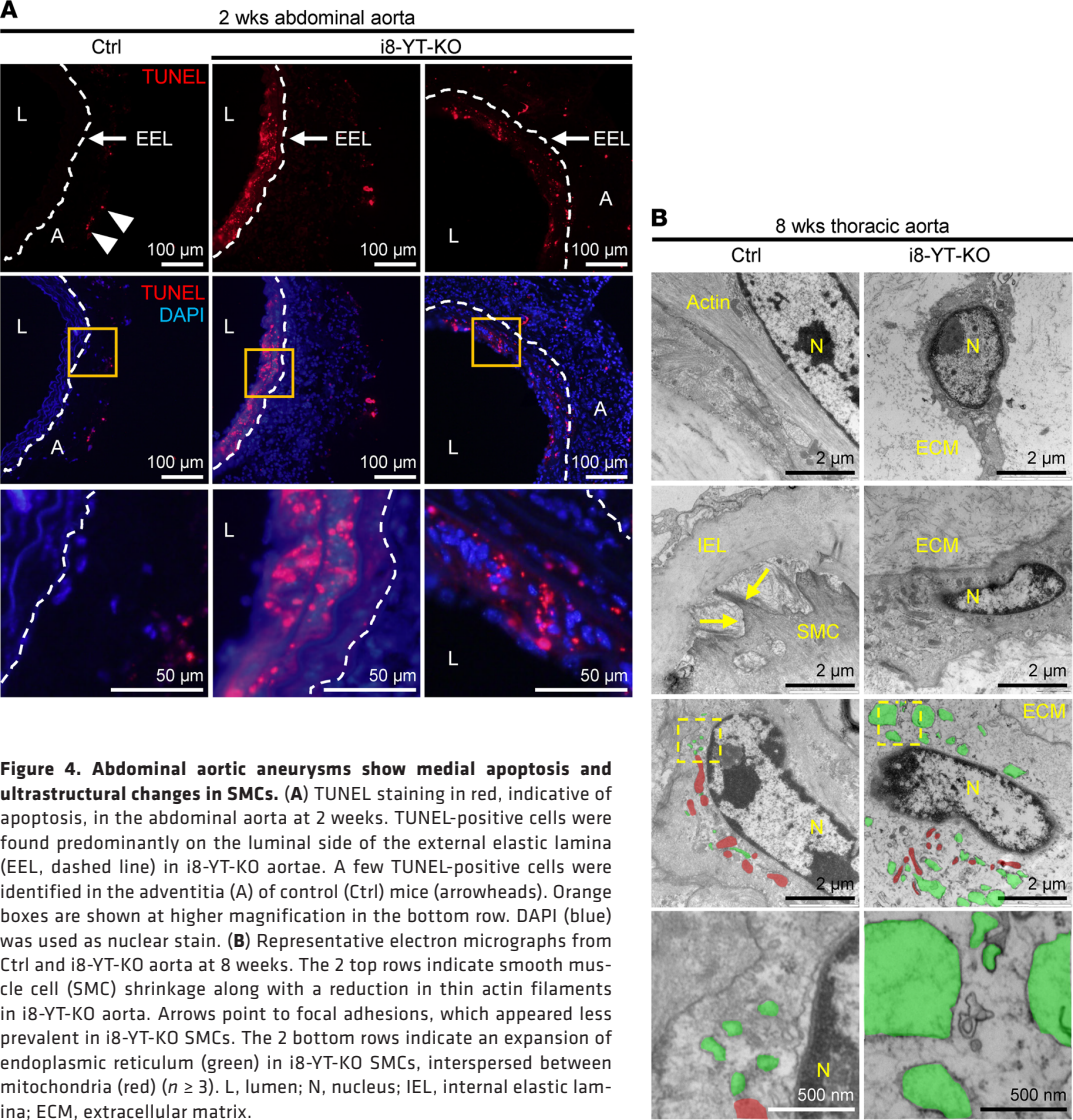
Abdominal aortic aneurysms show medial apoptosis and ultrastructural changes in SMCs. (**A**) TUNEL staining in red, indicative of apoptosis, in the abdominal aorta at 2 weeks. TUNEL-positive cells were found predominantly on the luminal side of the external elastic lamina (EEL, dashed line) in i8-YT-KO aortae. A few TUNEL-positive cells were identified in the adventitia (A) of control (Ctrl) mice (arrowheads). Orange boxes are shown at higher magnification in the bottom row. DAPI (blue) was used as nuclear stain. (**B**) Representative electron micrographs from Ctrl and i8-YT-KO aorta at 8 weeks. The 2 top rows indicate smooth muscle cell (SMC) shrinkage along with a reduction in thin actin filaments in i8-YT-KO aorta. Arrows point to focal adhesions, which appeared less prevalent in i8-YT-KO SMCs. The 2 bottom rows indicate an expansion of endoplasmic reticulum (green) in i8-YT-KO SMCs, interspersed between mitochondria (red) (*n ≥* 3). L, lumen; N, nucleus; IEL, internal elastic lamina; ECM, extracellular matrix.

To interrogate the nature and composition of aneurysms, we used Movat pentachrome, which stains several connective tissue elements. This revealed sparse SMC staining in the media of the i8-YT-KO abdominal and thoracic aortae (Supplemental Figure 1C), while blue staining, indicative of proteoglycan accumulation, was increased (Figure 3, A and B, and Supplemental Figure 4, A and B). In severe lesions, elastic lamellae were more widely spaced and fragmented in the abdominal aorta, and neointima formation was observed in 6 out of 7 analyzed mice (Figure 3, A and B). Moreover, the adventitia appeared thicker, with increased cellularity. Elastin disarray and the appearance of a thick neointima were also evident in lesions from the superior mesenteric artery (Figure 3C and Supplemental Figure 1C). The thickened media of the abdominal aorta displayed no significant change in number of elastic layers, supporting wider spacing of elastic lamella (Figure 3D). Apoptosis was supported by TUNEL staining, showing positive areas in both the media and neointima of severe lesions (Figure 4A). Apoptosis in the media of i8-YT-KO aortae was seen in 7 out of 34 tissue sections imaged, corresponding to 4 out of 5 of the i8-YT-KO mice analyzed. No control mice displayed similar apoptotic areas. Transmission electron microscopy revealed shrinkage of SMCs in the i8-YT-KO aorta, loss of actin filaments (Figure 4B, top 2 rows) and expansion of the endoplasmic reticulum (Figure 4B, bottom 2 rows; endoplasmic reticulum is highlighted in green and mitochondria in red). We also observed increased Ki67 staining indicative of proliferation in the thickened adventitia, but only occasionally in the media of aortae from i8-YT-KO mice (Supplemental Figure 4C).

**Figure 5:**
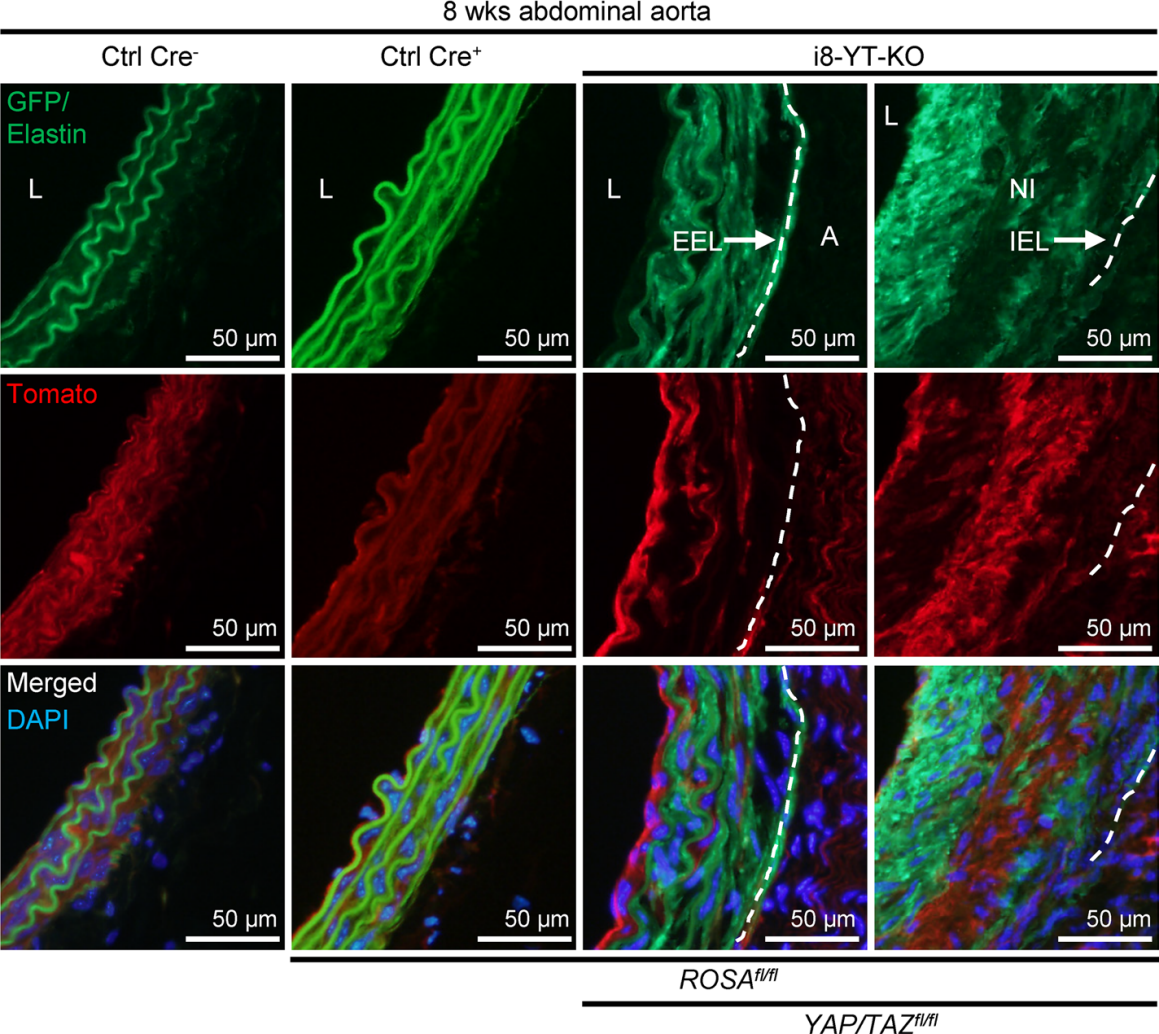
Lineage tracing in i8-YT-KO mice shows that SMCs contribute the bulk of cells to the neointima. i8-YT-KO mice on the mT/mG background were used to track cells derived from SMCs in aneurysms. mT/mG mice carry a transgene that expresses Tomato (red) before recombination. After recombination, the cells instead express GFP (green). The media stained red in control (Ctrl) Cre^–^ mice at 8 weeks after tamoxifen administration. The only green staining was from elastin autofluorescence. Ctrl Cre^–^ refers to Cre-negative tamoxifen-treated ROSA *Itga8-CreER^T2^* mice, whereas Ctrl Cre^+^ refers to Cre-positive tamoxifen-treated ROSA *Itga8-CreER^T2^* mice. Following recombination (i8-YT-KO), medial red staining was reduced, and green staining appeared between the autofluorescent lamellae. In advanced lesions with thick neointimas (NI) inside the internal elastic lamina (IEL, dashed line), the neointima was intensely green. This suggests that SMCs derived from the media contributed to the formation of neointima. In contrast, the adventitia (A) was largely GFP negative. The top right section is identical to the right panel in Figure 3B. DAPI (blue) was used as nuclear stain. L, lumen; EEL, external elastic lamina (dashed line).

i8-YT-KO mice also carrying the mT/mG Cre reporter were used for further characterization. Such mice express Tomato (red) prior to recombination, and this switches to GFP (green) in cells where Cre-mediated recombination occurred (Supplemental Figure 1B). Tomato fluorescence was higher in Ctrl Cre^–^ than in i8-YT-KO aorta, as expected (Figure 5). The only green fluorescence in Ctrl Cre^–^ aorta was that originating from elastic lamellae, which contributed to autofluorescence in both red and green channels (Figure 5, top row). In the i8-YT-KO aorta, interlamellar red fluorescence had switched to green. Importantly, neointimal areas were intensely green (Figure 5, fourth column), indicating that neointimal cells were, to a large extent, derived from SMCs. Comparably less green fluorescence was seen in the adventitia (Figure 5, third column), arguing that cells other than SMCs primarily contributed to adventitial expansion.

### Transcriptomic and proteomic surveys highlight additional phenotypes and plausible disease mechanisms.

**Figure 6:**
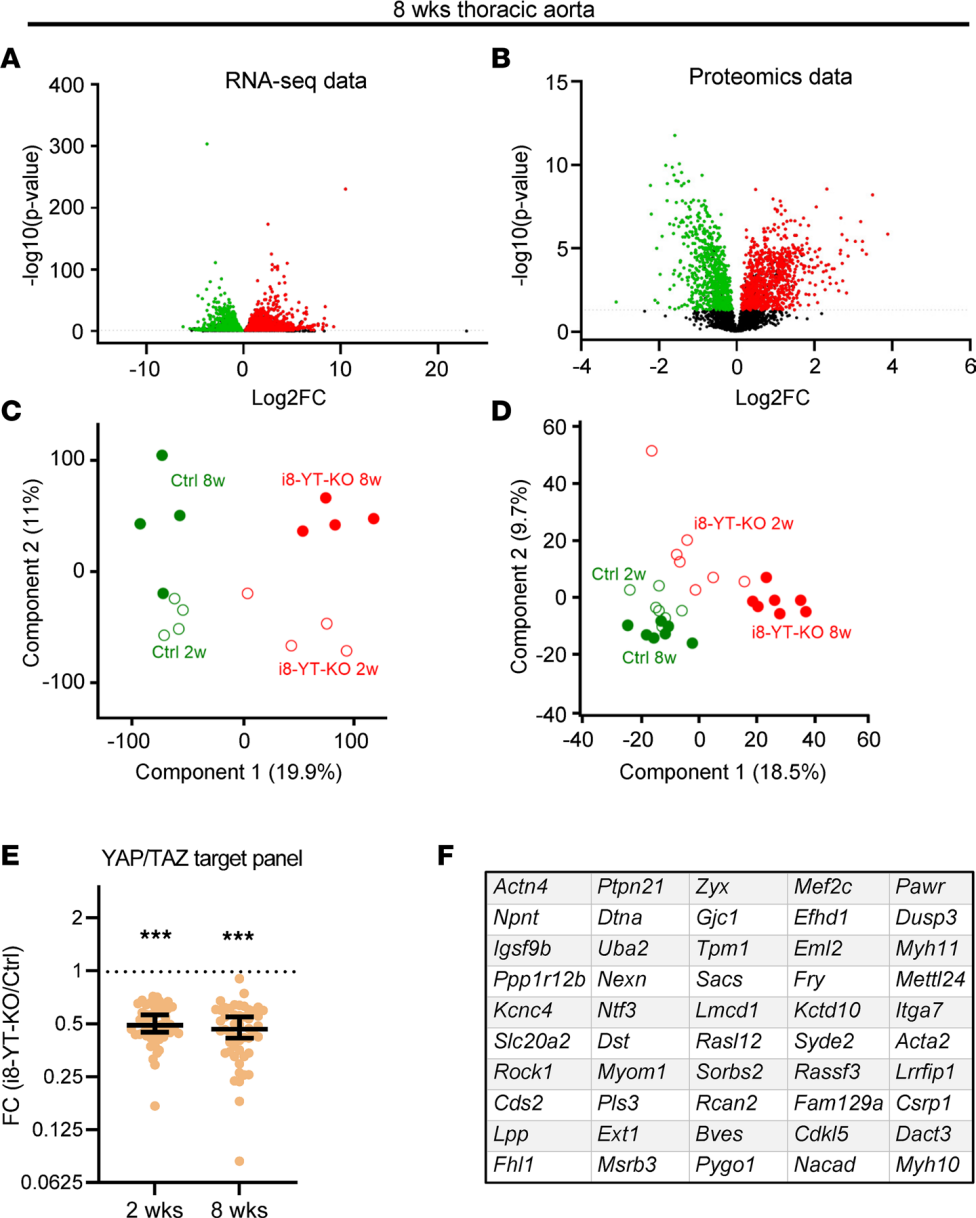
Transcriptomic and proteomic data highlight mechanisms of aneurysm formation in i8-YT-KO mice. mRNAs and proteins were assayed in thoracic aortae at 8 weeks after tamoxifen injections using RNA sequencing (*n* = 4) and mass spectrometry (*n* = 7). (**A** and **B**) Volcano plots for mRNA and protein data at 8 weeks. Below, principal component analysis (PCA) plots for transcriptomic (**C**) and proteomic (**D**) data at 2 and 8 weeks, respectively, are shown. Components 1 and 2 are presented in each. FC, fold change. (**E**) Demonstrates the effect of i8-YT-KO on the expression of 53 selected genes that are among the 1000 transcripts that correlate best with YAP1 in human aorta (GTExPortal) and that have been identified as potential YAP/TAZ targets in 2 independent studies (12, 13). (**F**) Using RNA-sequencing data from 2- and 8-week i8-YT KO aortae, the vascular smooth muscle YAP/TAZ target panel used in **E** was further refined to include the 50 genes shown. A 2-tailed Student’s *t* test with permutation-based FDR was used in **A** and **B**; Wilcoxon’s signed-rank test was used in **E**. ***P < 0.001.

To characterize molecular changes resulting from YAP/TAZ deletion in arterial SMCs, RNA sequencing and mass spectrometry analyses were conducted using aortae at 2 (Supplemental Figure 5) and 8 weeks after tamoxifen (Figure 6). Volcano plots revealed numerous upregulated and downregulated transcripts and proteins (Figure 6, A and B, and Supplemental Figure 5, A and B). Principal component analysis clearly separated the Ctrl and i8-YT-KO genotypes and ages along components 1 and 2, accounting for 18% to 19% variation of component 1 and 9% to 11% of component 2 (Figure 6, C and D). Using previously published RNA-sequencing data from *Myh11-Cre*-Y/T KO aorta (12, 13), we identified approximately 200 transcripts that were significantly downregulated in both data sets. These genes were then overlapped with the 1000 transcripts that correlated best with *YAP1* in human aorta, using the GTExPortal database (https://gtexportal.org/home/). This resulted in a list of 53 potential YAP/TAZ target genes (Supplemental Table 3). When RNA-sequencing data from i8-YT-KO aortae was compared with the YAP/TAZ target list, we found a significant downregulation of these genes at both the 2- and 8-week time points (Figure 6E). Adding the i8-YT-KO RNA-sequencing data, the list of YAP/TAZ targets could be further pruned to 50 genes that are significantly downregulated in all 4 data sets, and that overlap with genes that correlate with *YAP1* in humans (Figure 6F).

**Figure 7:**
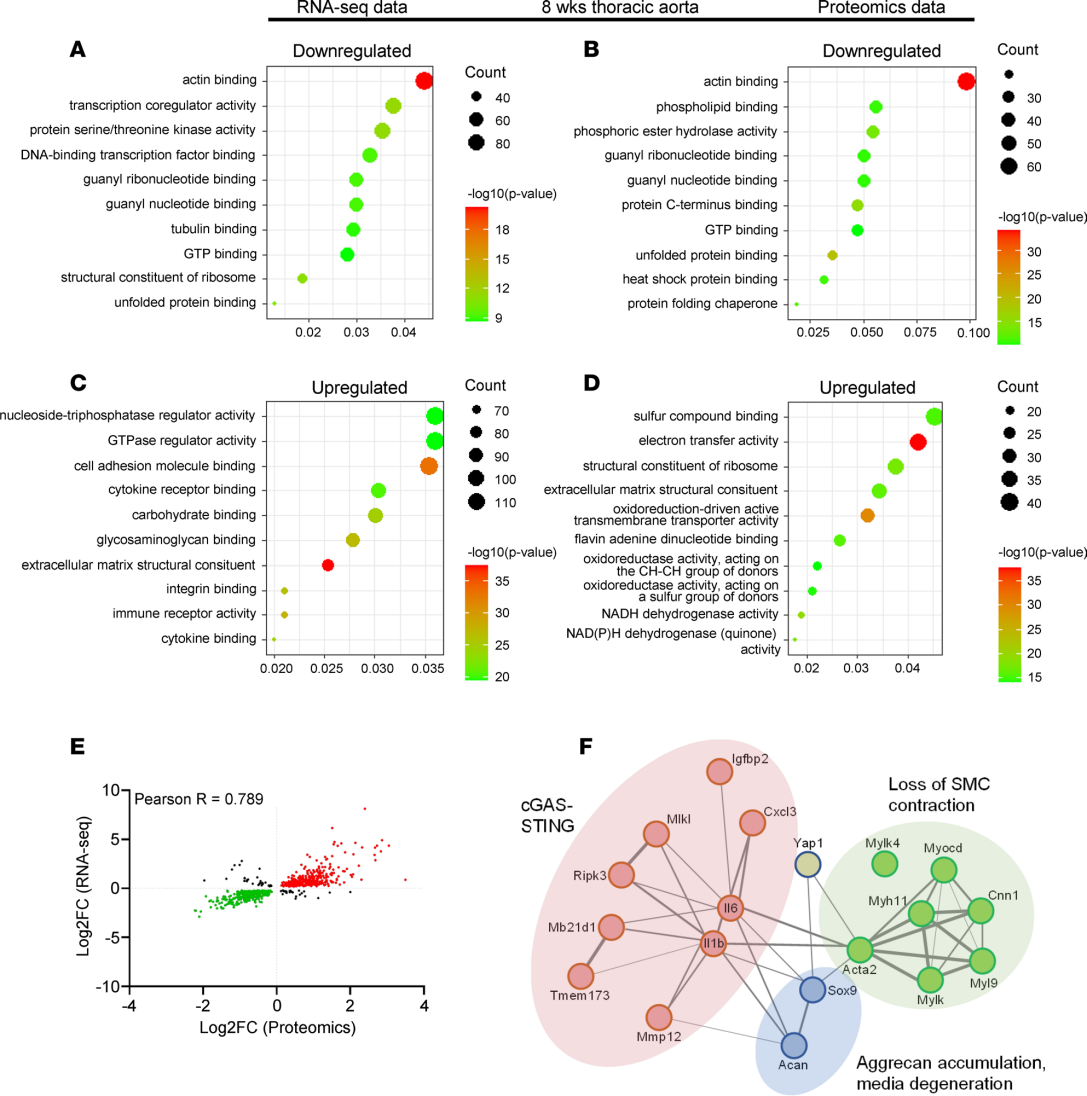
Transcriptomic and proteomic data highlight over- and underrepresented biological processes associated with aneurysm formation in i8-YT-KO mice. (**A**–**D**) Gene ontology enrichment analysis for downregulated transcripts (**A**) and proteins (**B**), as well as upregulated transcripts (**C**) and proteins (**D**) demonstrate significant pathways at 8 weeks. (**E**) Significant correlation between proteomic and transcriptomic data sets was seen. (**F**) STRING analysis that highlights 3 pathogenic domains of aneurysmal disease in i8-YT-KO mice. Pearson’s *R* was used in **E**. FC, fold change.

Gene ontology enrichment analysis highlighted “actin binding” as the most significant category for reduced transcripts and proteins at both 2 and 8 weeks (Figure 7, A and B, and Supplemental Figure 5, C and D). The categories “GTP binding” and “unfolded protein binding” were also represented in both transcriptomic and proteomic data. The most significant categories of transcripts and proteins that increased are shown in Figure 7, C and D, and Supplemental Figure 5, E and F. We noted that RNA sequencing had captured inflammatory mediators and transcription factors, including myocardin (*Myocd*, reduced) and *Sox9* (increased), that were not captured by proteomics. We attribute these differences to the protein extraction protocol and note that the overall correlation between transcripts and proteins was excellent, with a Pearson’s correlation coefficient of 0.79 (Figure 7E and Supplemental Figure 5G). This strongly cross-validates both analyses.

STRING (search tool for the retrieval of interacting genes/proteins) analysis was performed by manually selecting genes that were significantly dysregulated in the RNA-sequencing analysis, and that have been previously described to be associated with YAP pathways (2, 5, 13). The resulting STRING network highlights protein interactions between 3 selected clusters that were studied further below: “loss of SMC contraction,” “cGAS/STING,” and “aggrecan accumulation and media degeneration” (Figure 7F).

### Loss of SMC contractility.

**Figure 8:**
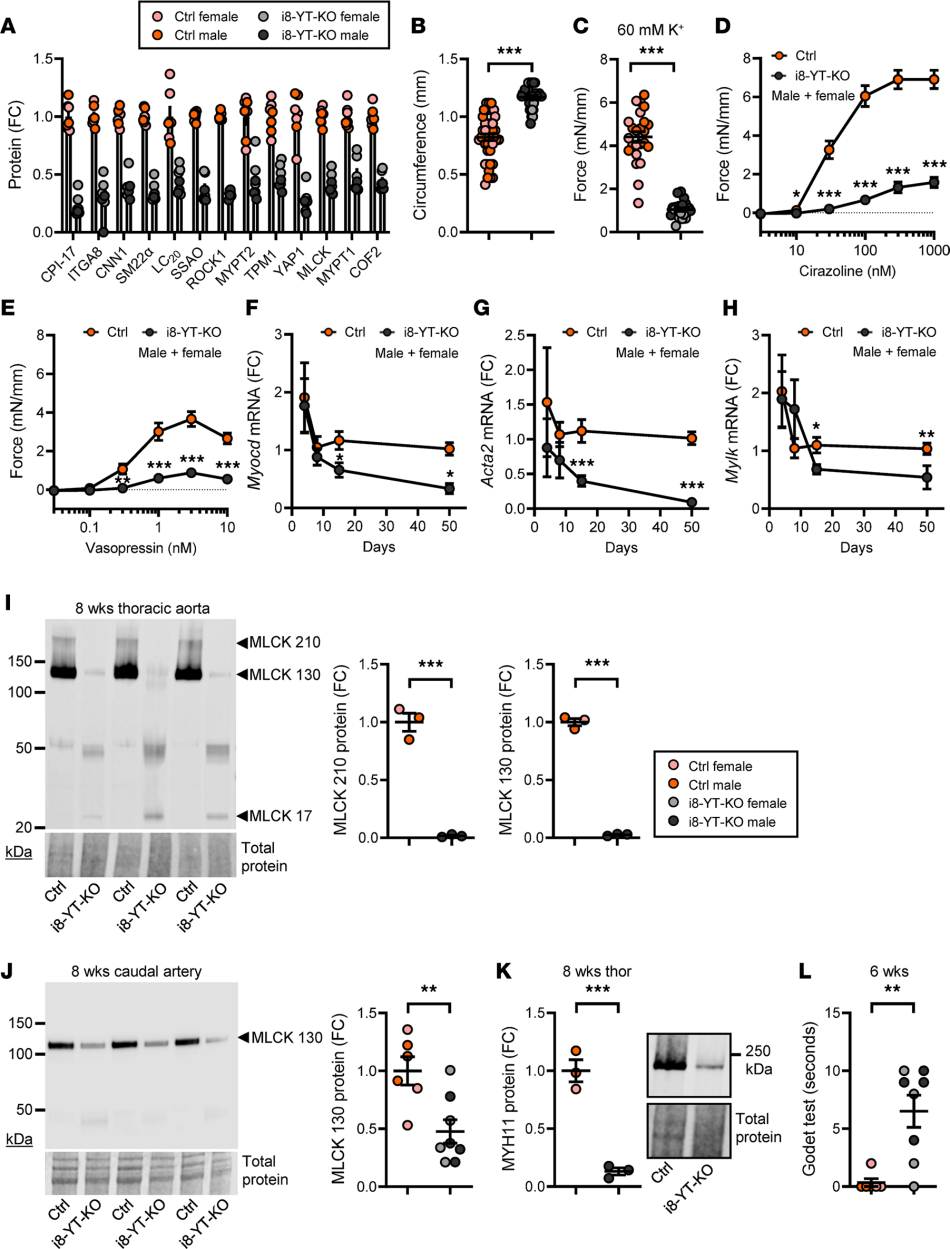
Loss of SMC differentiation and arterial contractility in i8-YT-KO mice at 8 weeks. (**A**) Proteins with a role in SMC contraction that were significantly reduced in the proteomic experiment at 8 weeks after tamoxifen. Several of these, including integrin α8 (ITGA8), calponin-1 (CNN1), transgelin (SM22α), membrane primary amine oxidase (SSAO), and myosin light chain kinase (MLCK), are targets of the transcription factor myocardin (Myocd), the master regulator of SMC differentiation. To examine contractility, we mounted caudal arteries in Mulvany myographs and applied a basal tension of 5 mN. FC, fold change. (**B**) Shows that the internal circumference was greater in i8-YT-KO mice compared with controls (Ctrl) at 5 mN (*n ≥* 18 mice and 38 arteries). (**C**–**E**) Following equilibration, arteries were stimulated with 60 mM K^+^ (**C**, *n ≥* 18 mice and 38 arteries), the α1-adrenergic receptor agonist cirazoline (**D**, *n ≥* 10 mice and 20 arteries), and vasopressin (**E**, *n ≥* 10 mice and 20 arteries). Preparations were washed and maintained in a relaxed state for 25 minutes between stimuli. Transcriptomic data indicated reduced expression of *Myocd* at 8 weeks after tamoxifen. (**F**) This reduced expression was confirmed using RT-qPCR in time-course studies of the aorta (*n ≥* 4). (**G** and **H**) Parallel reduction of *Acta2* (**G**, *n ≥* 4) and *Mylk* (**H**, *n ≥* 4) was observed. (**I**) Western blot for MLCK using 8-week aortae along with quantification of the bands at 210 and 130 kDa (*n* = 3). (**J**) MLCK in caudal arteries at 8 weeks (*n ≥* 6). (**K**) Reduction in smooth muscle myosin heavy chain (MYH11) was also confirmed by Western blotting (*n* = 3). (**L**) Results from the Godet test (*n ≥* 6). This test measures time taken (in seconds) for skin to rebound from pitting and is considered an indication of edema. *P < 0.05; **P < 0.01; ***P < 0.001 by Mann-Whitney test (**B** and **L**), 2-tailed Student’s *t* test (**C** and **F**–**K**), or 2-way ANOVA with Bonferroni’s post hoc test (**D** and **E**).

RNA-sequencing data showed reduction in the transcription factor *Myocd* and several of its direct targets, including *Acta2*, *Myh11*, *Tagln*, and *Tpm1*. Contractile proteins were also reduced in the proteomic analysis (Figure 8A; 8 weeks, *P* < 0.05 for all), although myocardin was not detected. This favored loss of SMC differentiation and reduced arterial contractility, which could contribute to an increased arterial diameter and increased wall stress. Because the aorta was invariably affected by lesions, we used the caudal artery, which only rarely had visible aneurysms, to assess contractility. Caudal artery segments obtained 8 weeks after tamoxifen treatment were mounted in myographs, and 5 mN of passive force was applied in relaxing physiological buffer. The circumference at this precise force was greater in i8-YT-KO arteries than in controls (Figure 8B and Supplemental Figure 6), extending similar findings by ultrasound and histology of the aorta. After equilibration, arteries were stimulated to contract using depolarizing buffer (60 mM K^+^) and the α_1_-receptor agonist cirazoline and vasopressin. Contraction for all modes of activation was severely blunted (Figure 8, C–E and Supplemental Figure 6). We also examined arteries in myographs at 2 weeks and found that circumference at 5 mN was increased along with a rightward shift of the circumference-tension relationship, but contractility remained unchanged or was even increased (Supplemental Figures 6 and 7, A–E).

Antibodies for myocardin are poor (25); therefore, we confirmed its reduction by RT-qPCR in time-course experiments. *Myocd* was significantly reduced at 2 weeks and onwards (Figure 8F), and this was associated with a parallel reduction in the archetypal target gene *Acta2* (Figure 8G, see Supplemental Figure 7F for 2-week data). *Mylk*, which encodes myosin light chain kinase (MLCK), was reduced in both transcriptomic and proteomic experiments, and this was independently confirmed by RT-qPCR (Figure 8H, see Supplemental Figure 7F for 2-week data), and by Western blotting in 8-week aortae (Figure 8I) and caudal artery (Figure 8J). The anti-MLCK antibody–labeled bands at 210 and 130 kDa were reduced (Figure 8I), while bands at around 50 and 20 kDa were increased in i8-YT-KO aortae. The bands at 210 and 130 kDa represent long MLCK and smooth muscle MLCK, respectively, while the lower bands may represent telokin-like peptides derived from the 3′ end of the *Mylk* gene through alternative transcription start sites. Immunofluorescent staining (Supplemental Figure 7G) and Western blotting at 2 weeks (Supplemental Figure 7H) confirmed a reduction in smooth muscle MLCK at this earlier time in both abdominal and thoracic aortae. No change in MLCK staining was apparent in the caudal artery at 2 weeks (Supplemental Figure 7I), even if a clear-cut reduction was manifest at 8 weeks (Figure 8J). This suggested faster MLCK repression in aorta versus distal arteries. Reduction of *Myh11*, which encodes smooth muscle myosin, was also confirmed by Western blotting using 8-week thoracic aorta (Figure 8K).

Considering everything else being equal, a reduction in arterial resistance will increase hydrostatic capillary pressure and thereby promote edema. In i8-YT-KO mice, blood pressure was reduced, but combined changes in arterial diameter and contractility appeared greater. We therefore predicted edema and tested this by measuring the recovery time after skin pitting (Godet test, 6 weeks). Recovery time was significantly increased in i8-YT-KO compared with Ctrl mice (Figure 8L). Another prediction from a reduced arterial resistance was a decreased end-systolic volume, and this was confirmed by echocardiography (Supplemental Table 2). This occurred at a maintained cardiac output and heart rate (Supplemental Table 2). In all, these studies therefore supported reduction of peripheral arterial resistance due to reduced contractile differentiation and outward remodeling in i8-YT-KO mice at 3 to 8 weeks after tamoxifen administration.

### A proinflammatory switch and accumulation of immune cells in the i8-YT-KO aorta.

**Figure 9:**
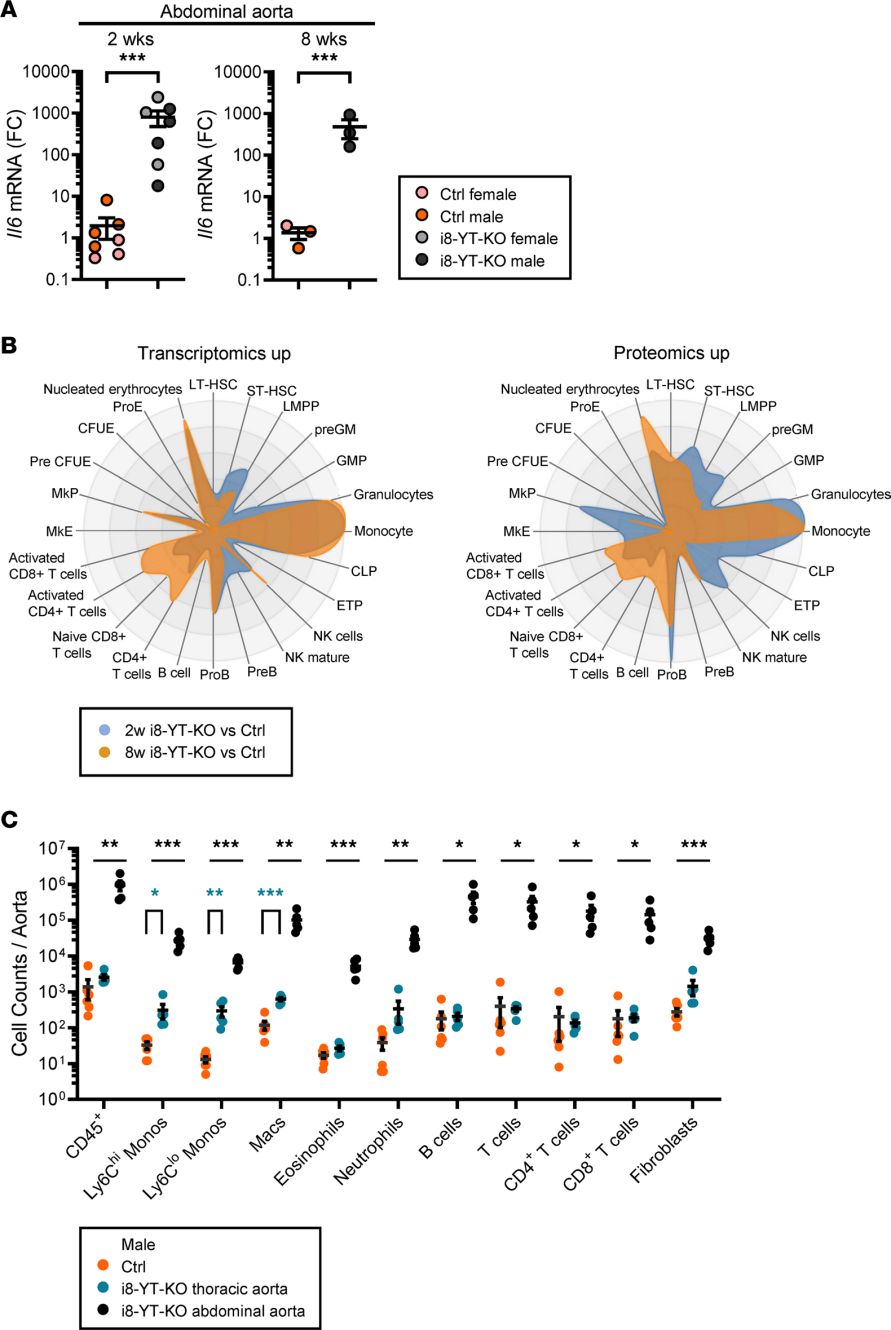
Aortic inflammation in i8-YT-KO mice involves induction of proinflammatory mediators and infiltration of several immune cell populations. RNA sequencing indicated upregulation of numerous inflammatory mediators in the aorta. (**A**) RT-qPCR assays for the proinflammatory cytokine interleukin 6 (*Il6*) confirmed upregulation in the abdominal aorta (2 weeks: *n* = 7; 8 weeks: *n* = 3). FC, fold change. (**B**) Upregulated transcripts and proteins were next used to predict distribution of bone marrow–derived inflammatory cells using CellRadar. Irrespective of the data set, monocytes/macrophages and granulocytes were predicted to reside in knockout (i8-YT-KO) aortae. (**C**) To directly measure infiltration of immune cells, cells were isolated from the aorta (8 weeks) and separated by flow cytometry. We assayed the thoracic (blue) and abdominal (black) aortae separately in i8-YT-KO mice, and compared cell counts with those of the whole aorta in control (Ctrl) mice (orange). All immune cell populations were increased in the abdominal aorta, whereas only monocytes and macrophages were significantly increased in thoracic aorta. LT-HSC, long-term hematopoietic stem cells; ST-HSC, short-term hematopoietic stem cells; LMPP, lympho-myeloid primed progenitor; GM, granulocyte-macrophage; GMP, granulocyte-monocyte progenitor; CLP, common lymphoid progenitor; ETP, early T cell precursor; NK, natural killer; MkE, megakaryocyte/erythroid; MkP, megakaryocyte progenitor; CFUE, colony-forming unit-erythroid; ProE, pro-erythrocyte. *P < 0.05; **P < 0.01; ***P < 0.001 by 2-tailed Student’s *t* test (**A** and **C**).

**Figure 10:**
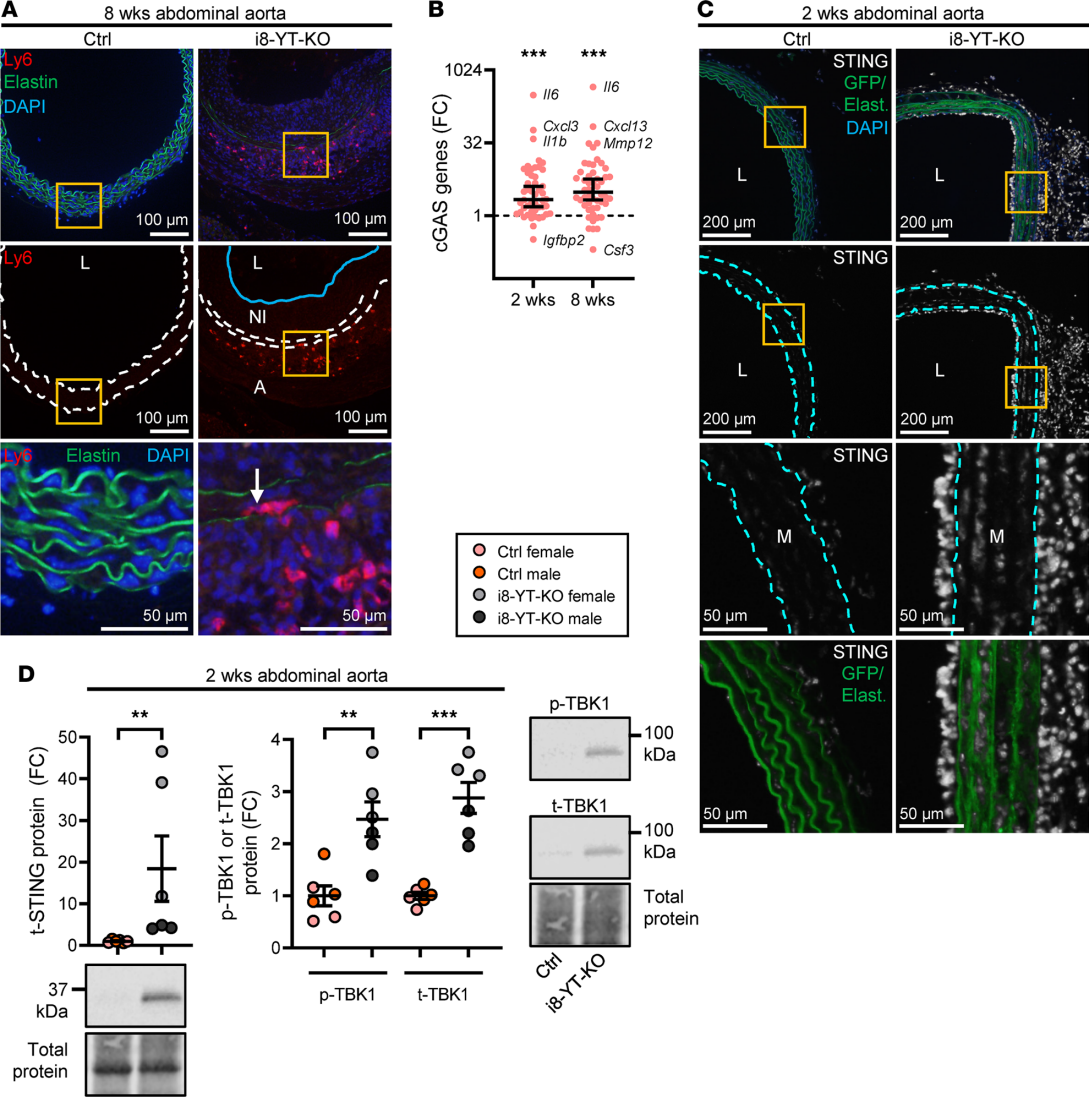
Aortic inflammation in i8-YT-KO mice involves cGAS genes. (**A**) We used Ly6 immunofluorescence to localize monocytes in aneurysms and found them predominantly in the adventitia. Arrow depicts rare monocytes infiltrating the media. Dashed lines represent the internal and external elastic lamina and the blue line demarcates neointima border. NI, neointima; L, lumen; A, adventitia. (**B**) To approach possible underlying mechanisms of inflammation and immune cell infiltration, we interrogated our transcriptomic data sets with a panel of cGAS/STING target genes. This panel was significantly increased at both time points (2 and 8 weeks), with the largest relative changes seen for *Il6*, *Cxcl3*, *Il1b*, and *Mmp12*. FC, fold change. (**C**) mT/mG mice were used to stain for STING (grayscale), which was high in the neointima and in the adventitia. However, smooth muscle cells (SMCs) in media also showed clear evidence of STING induction. Boxed areas are highlighted in magnified images (third and fourth rows). Some STING-positive cells were GFP positive (green, bottom row), suggesting that they are of SMC origin. Dashed lines represent the internal and external elastic lamina. (**D**) We next assayed the level of total (t)-STING by Western blotting (*n* = 6) and found it to be increased. Also shown are the Western blot data for p-TBK1 and t-TBK1 in Ctrl and i8-YT-KO aortae (*n* = 6). t-STING, p-TBK1, and t-TBK1 were blotted for on the same membrane. The membrane was stripped of anti–p-TBK1 before being blotted and analyzed for t-TBK1. DAPI (blue) was used as nuclear stain. **P < 0.01; ***P < 0.001 by Wilcoxon’s signed-rank test (**B**), Mann-Whitney test (**D**, left graph), or 2-tailed Student’s *t* test (**D**, right graph).

Our RNA-sequencing analysis revealed increases in numerous inflammatory mediators, and an almost 1000-fold increase in *Il6* was confirmed by RT-qPCR at 2 and 8 weeks (Figure 9A and Supplemental Figure 8A). We hypothesized that aneurysm formation may associate with immune cell infiltration and approached this using the CellRadar tool (https://karlssong.github.io/cellradar/). CellRadar uses sets of cell lineage markers to predict enrichment of cells derived from the bone marrow. We focused on transcripts and proteins that increased in the transcriptomic and proteomic data sets. CellRadar predicted monocytes and granulocytes in i8-YT-KO aortae, as shown by the protruding areas to the right in Figure 9B. An increase in lymphoid lineages between 2 and 8 weeks was also suggested. To directly test these predictions, we dissociated aortic cells from 8-week mice and subjected them to flow cytometry. We separated the i8-YT-KO aortae into abdominal and thoracic parts and compared their immune cell infiltration with that in full-length aortae from Ctrl mice. All immune cell subpopulations were increased in the abdominal i8-YT-KO aorta compared with Ctrl, including CD45^+^ leukocytes, inflammatory Ly6C^hi^ and patrolling Ly6C^lo^ monocytes, macrophages, eosinophils, neutrophils, B cells, total T cells, as well as both CD4^+^ and CD8^+^ T cell subpopulations (Figure 9C, black versus orange symbols). Monocytes and macrophages were increased in the thoracic i8-YT-KO aorta (Figure 9C, blue versus orange symbols). Inflammatory monocyte and neutrophil percentages were increased in the blood and spleen of i8-YT-KO mice, suggesting increased myeloid cell production and release into the circulation (Supplemental Figure 8, B–D). To localize infiltrating immune cells, we stained for monocytes (Figure 10A) and macrophages (Supplemental Figure 9A), and could localize the former to the aortic adventitia, while the latter were present in both the adventitia and neointima. Together, these findings established immune cell infiltration into the i8-YT-KO aorta.

With previous work (5) suggesting unscheduled activation of cGAS/STING as a mechanism for inflammation after SMC depletion of YAP/TAZ, we used a panel of cGAS/STING target genes (20) for interrogation of our RNA-sequencing data set. Fold-changes in cGAS/STING target genes in the i8-YT-KO aorta deviated significantly and positively from the median of the entire data set (Wilcoxon’s 1-sample test) at both times (Figure 10B). We also found that *Sting1* (*Tmem173*) was increased at the mRNA (Supplemental Figure 9B) and protein level (Figure 10C, D). Using mT/mG mice and STING staining, we could show that a part of its induction occurred in SMCs (Figure 10C), even if the major increase at this time was in the adventitia and neointima. STING activation leads to phosphorylation of the kinase TBK1, which promotes NF-κB–driven transcription of inflammatory mediators. We thus assayed phosphorylated (S172) and total TBK1 using specific antibodies and Western blotting. Phosphorylated (p-TBK1) levels were increased in abdominal aorta, as were total (t-TBK1) levels (Figure 10D). In 2-week thoracic aorta, on the other hand, p-TBK1 was increased without an attendant increase in t-TBK1 (Supplemental Figure 9C). Thus, aortic p-TBK1 increases in i8-YT-KO mice. In all, these findings demonstrate upregulation of a large battery of inflammatory mediators in the aorta, along with infiltration of innate immune cells. STING-TBK1 may contribute to inflammation, and TBK1 activation (increased p-TBK1 vs. t-TBK1 ratio) is seen in the thoracic aorta at 2 weeks.

### Loss of YAP and TAZ in aortic SMCs promotes chondrogenic differentiation.

**Figure 11:**
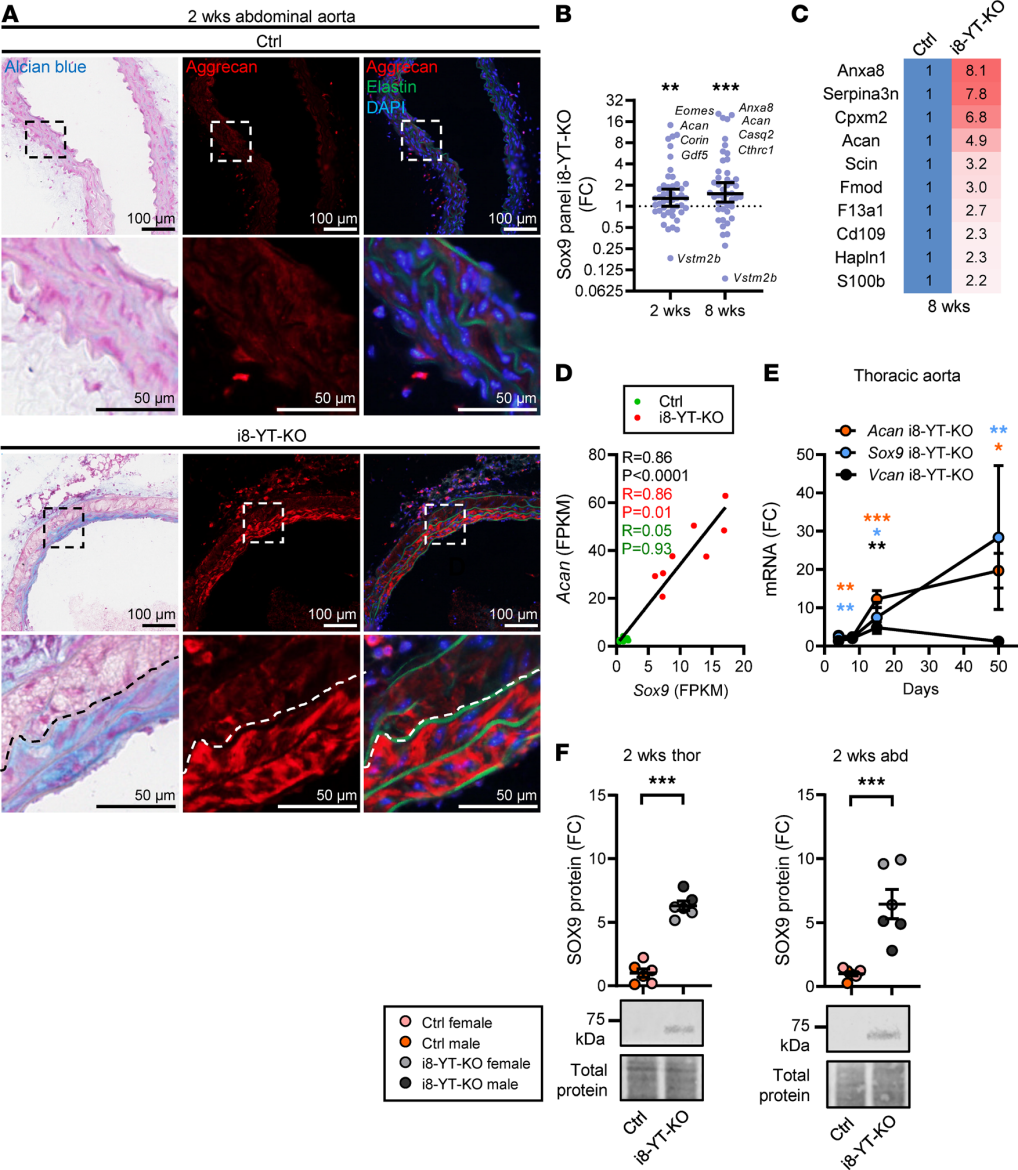
SOX9 is induced and drives a program of chondrogenic differentiation in i8-YT-KO aortae. (**A**) Staining of abdominal aorta with Alcian blue (left) and with an antibody directed against aggrecan (middle and right) in consecutive sections. Areas that were blue overlapped with areas that stained positive for aggrecan in red. Our transcriptomic data sets indicated increased expression of the chondrogenic transcription factor *Sox9*, a known regulator of aggrecan. (**B**) We thus interrogated RNA-sequencing data with a panel of knockout-validated SOX9 target genes and found the panel to be increased at 2 weeks and 8 weeks. FC, fold change. (**C**) A larger panel of SOX9 target genes in cartilage was next overlaid with our 8-week proteomic data, and the SOX9-regulated proteins that increased >2-fold are listed. (**D**) *Sox9* and aggrecan (*Acan*) correlate in our transcriptomic data sets across genotypes and over time. Correlations were also tested for i8-YT-KO mice and control (Ctrl) mice separately, and the *R* values of those tests are given in red and green. (**E**) RT-qPCR for *Sox9*, aggrecan (*Acan*), and versican (*Vcan*) confirmed upregulation in thoracic aortae (*n ≥* 4). (**F**) Western blot data for SOX9 in thoracic and abdominal aortae (*n ≥* 6). DAPI (blue) was used as nuclear stain. *P < 0.05; **P < 0.01; ***P < 0.001 by Wilcoxon’s signed-rank test (**B**), Spearman’s correlation (**D**), or 2-tailed Student’s *t* test (**E** and **F** [except for versican, 8 weeks in panel **E**, where Mann-Whitney was used]).

Movat pentachrome consists of 5 different staining reagents, one of which is Alcian blue, a stain that is used to visualize acidic polysaccharides such as glycosaminoglycans and proteoglycans. To investigate the occurrence of proteoglycans in the aortic media, we stained cryosections of aorta with Alcian blue. Nuclei were counterstained with Fast Red. Alcian blue staining was increased in i8-YT-KO compared with Ctrl aortae (Figure 11A, left column). By transcriptomic and proteomic analyses of the 20 proteoglycans that make up the aortic proteoglycanome (defined by Cikach et al., ref. 26), we could determine that aggrecan was the most dramatically and consistently increased proteoglycan in i8-YT-KO aortae. Alcian blue and aggrecan staining in consecutive sections demonstrated considerable overlap in i8-YT-KO aortae (compare middle and left in Figure 11A, bottom).

The transcription factor SOX9 is an important direct driver of aggrecan expression in chondrocytes (27), and *Sox9* was increased in our RNA-sequencing experiment at 2 and 8 weeks. We thus interrogated our transcriptomic data with a panel of KO-validated SOX9 target genes (28), and found the panel to be increased at 2 and 8 weeks (Figure 11B). Moreover, when intersecting our 8-week proteomic data with a list of cartilage SOX9 targets (29), we found several that were increased. Aortic SOX9 targets with greater than 2-fold changes at the protein level are given in Figure 11C. Strikingly, *Sox9* and *Acan* correlated across all time points and genotypes in our RNA-sequencing data (Figure 11D, black). Significant correlation was also seen when only i8-YT-KO aortae were considered (Figure 11D, red), but not when only Ctrl aortae were considered (Figure 11D, green). RT-qPCR confirmed time-dependent *Sox9* and *Acan* accumulation in i8-YT-KO thoracic aortae (Figure 11E), whereas versican (*Vcan*) only increased at 2 weeks. The response was rapid, occurring within 7 days of the first tamoxifen injection (Figure 11E). Western blotting demonstrated greater than 6-fold increases in the SOX9 protein in thoracic and abdominal KO aortae at 2 weeks (Figure 11F). Taken together, these findings argue that increased SOX9 expression in the i8-YT-KO aorta promotes a program of chondrogenic differentiation that involves aggrecan accumulation.

**Figure 12:**
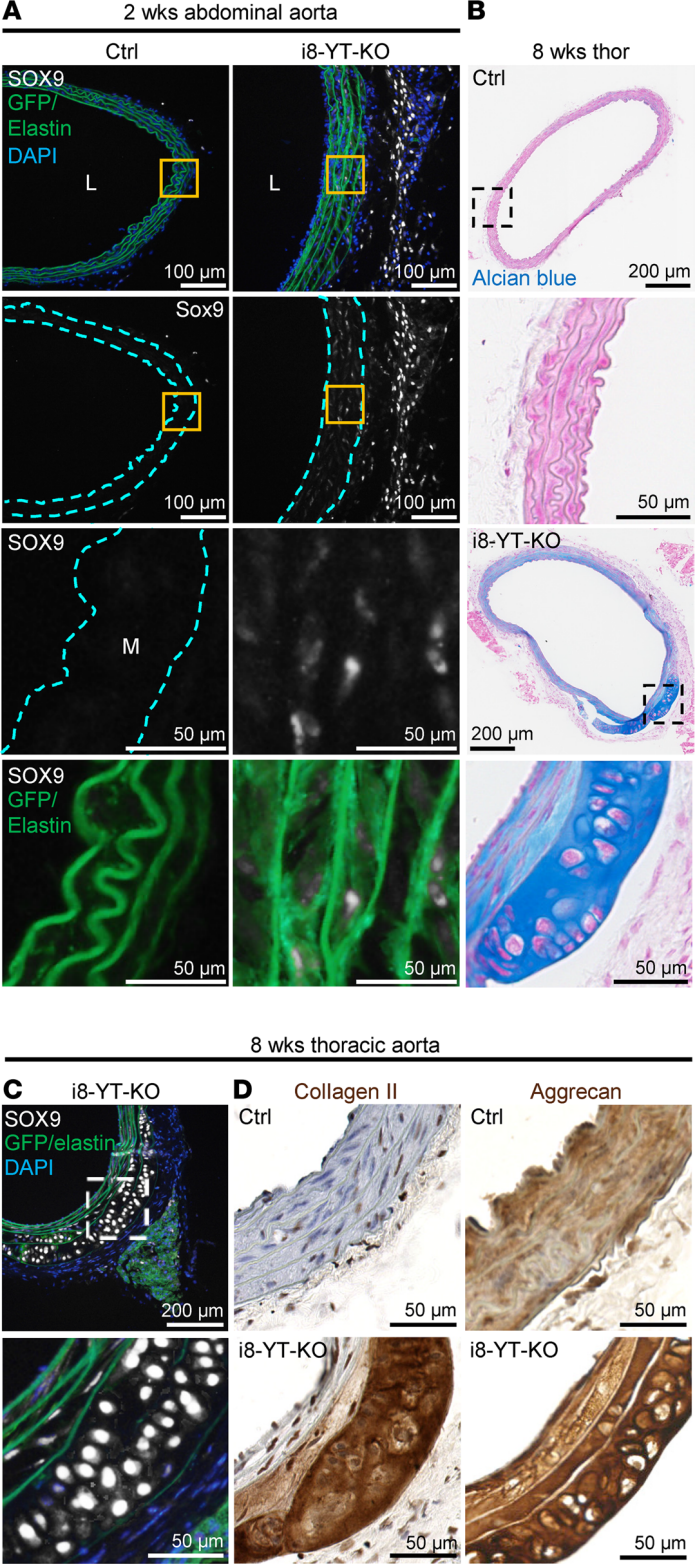
SOX9 is induced in i8-YT-KO aortae and drives a program of chondrogenic differentiation. (**A**) Staining for SOX9 (grayscale) in mT/mG control (Ctrl) and i8-YT-KO mice. No SOX9-positive cells were observed in Ctrl aortae. In contrast, SOX9-positive cells resided both in the adventitia and in the media (M) of the i8-YT-KO aortae. Those in the media are highlighted in the magnified images (third row, orange boxes in the low magnifications above). SOX9-positive cells in the media were GFP positive (green, bottom row), showing that they were of smooth muscle origin. (**B**) Alcian blue staining of cartilage-like tissue in the outer layers of the media in i8-YT-KO mice. (**C** and **D**) The same tissue stained strongly for SOX9, collagen II, and aggrecan, and it was demarcated by elastic lamellae. DAPI (blue) was used as nuclear stain. L, lumen.

To examine whether SOX9 increases specifically in SMCs, we used mT/mG i8-YT-KO mice and stained for SOX9 (Figure 12A). SOX9 staining was increased in the adventitia and media (Figure 12A, grayscale). Quantification showed that the SOX9-positive area increased from 0 ± 0 to 1.9% ± 0.8% (*P* < 0.01, *n* = 3). High-magnification images showed that SOX9-positive cells in the media were GFP positive (Figure 12A, bottom), arguing that SMCs contribute to the increase in SOX9. In further support of this notion, we identified instances where apparent cartilage accumulated in the i8-YT-KO aorta (Figure 12B). This cartilage constituted an integral part of the media, was highly basophilic, and the resident cells resided in lacunae, as they do in true cartilage (Figure 12B, bottom). The same area stained strongly for SOX9 (Figure 12C), cartilage collagen (collagen II, *Col2a1*), and aggrecan (Figure 12D). In these extreme examples of chondrogenic differentiation, the cartilage was demarcated by elastic lamellae on both sides, suggesting initiation of the process inside the media. Taken together, these findings argued that loss of YAP/TAZ promotes a program of chondrogenic differentiation in SMCs.

### Sox9 induction represents the earliest detectable change in the i8-YT-KO aorta.

**Figure 13:**
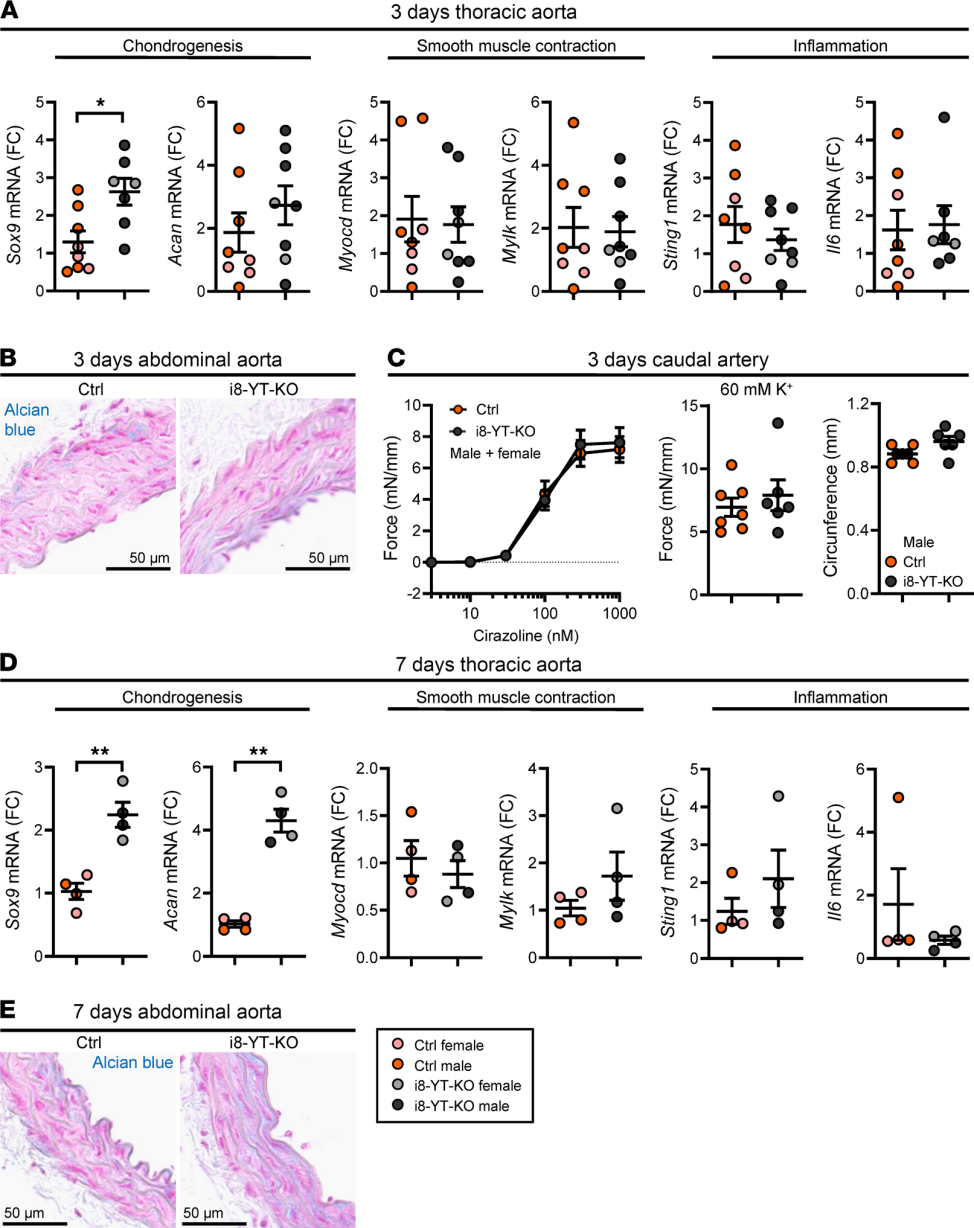
*Sox9* induction is the earliest detectable change in the i8-YT-KO aorta. This study identified 3 areas of aneurysm pathogenesis in i8-YT-KO mice, involving SOX9 and chondrogenesis, myocardin (Myocd) and contractile differentiation, and STING and inflammation. To examine which comes first, we analyzed mice at 3 (**A**–**C**) and 7 days (**D** and **E**) following the first injection of tamoxifen. We assayed transcripts relevant for each of the 3 areas of pathogenesis using RT-qPCR. (**A** and **B**) *Sox9* was the only transcript that was increased at 3 days. A single outlier was identified using the iterative Grubb’s method and excluded. At 3 days, *Myocd* and *Sting1* were unchanged (*n* = 7) (**A**), and no difference in Alcian blue staining was seen (**B**). FC, fold change. (**C**) Loss of contractility and remodeling of the caudal artery had not yet occurred (*n ≥* 4 mice and 6 arteries). (**D** and **E**) At 7 days, both *Sox9* and *Acan* were increased (**D**), while the remainder of the transcripts remained inert (*n* = 4) and Alcian blue staining had not yet followed suit (**E**). *P < 0.05; **P < 0.01 by 2-tailed Student’s *t* test (**A**, **C** [right 2 graphs], and **D** [except *Il6*, where Mann-Whitney was used]) or 2-way ANOVA with Bonferroni’s post hoc test (**C**, left graph).

The current work focused on 3 major areas of aortic pathology, involving reduced SMC differentiation, a proinflammatory transition in the aortic wall, and SOX9-driven chondrogenic differentiation. We hypothesized that chondrogenic differentiation precedes other phenotypes. To test this, we isolated aortae at 3 and 7 days after KO induction (counted from the first injection of tamoxifen) and assayed representative transcripts by RT-qPCR. We found that *Sox9* increased at 3 days, whereas *Acan,* *Myocd*, *Mylk*, *Sting1*, and *Il6* remained unchanged (Figure 13A). No apparent change in Alcian blue staining, contractile function, or vascular remodeling was observed at this time point (Figure 13, B and C). At 7 days (Figure 13D), *Sox9* mRNA was still increased, and *Acan* had followed suit, but *Myocd*, *Mylk*, *Sting*, and *Il6* remained unchanged. The increased *Acan* expression had not yet translated into increased Alcian blue staining at this time point (Figure 13E). *Sox9* induction in i8-YT-KO aortae therefore represents the earliest aortic change among those examined.

### YAP1, contractile, inflammatory, and chondrogenic associations in human arteries.

To increase the translational value of our findings, we lastly examined whether YAP and TAZ associate with contractile differentiation, inflammation, and chondrogenic differentiation in human arteries. For this we correlated YAP with all other transcripts in 3 arteries from an RNA-sequencing database for human organs (GTExPortal) and examined *R* values for (a) a panel of MYOCD target genes, (b) a panel of STING–NF-κB target genes, and (c) a panel of SOX9 target genes. The MYOCD panel *R* values deviated positively and significantly from the median of the entire data set (Supplemental Figure 10A), arguing that YAP promotes contractile differentiation in human arteries. Conversely, the STING–NF-κB panel deviated negatively and significantly from the median (Supplemental Figure 10B), arguing that YAP prevents unscheduled activation of proinflammatory signaling in human arteries. No significant deviation was seen for the SOX9 panel *R* values (Supplemental Figure 10C), but we note that the database includes grossly normal tissues, and we had failed to see a correlation between *Sox9* and *Acan* when Ctrl mice were considered in isolation (c.f. Figure 11D, green). Exemplary correlations are shown in Supplemental Figure 10, D–F, and while the group of SOX9 target genes failed to show an association, some individual targets were negatively correlated with YAP (Supplemental Figure 10F). These findings were further supported by a stratification approach, comparing individuals with high and low arterial YAP expression (Supplemental Figure 10, G–I).

## Discussion

In the present body of work, we generate and present a mouse model that develops spontaneous aortic aneurysms while evading a severe visceral myopathy seen in other adult SMC KOs for YAP and TAZ (12, 13). In contrast to the i8-YT-KO mouse, genetically determined mouse models of aneurysm formation are commonly hypertensive and/or hyperlipidemic (30). Only a few normotensive and normolipidemic mouse models exist, including the Blotchy mouse and mice deficient for lysyl oxidase (Lox) that are unable to crosslink elastin and collagen (31, 32). Mice deficient for TIMP-1 (tissue inhibitor of metalloproteinase-1) also develop aneurysms but only upon saline or elastase infusion and not spontaneously (33).

We present challenging findings in relation to the temporal order of change in the aortic wall during aneurysm progression. The 3 major areas of vascular pathology that we have investigated relate to loss of SMC differentiation, aortic inflammation, and accumulation of proteoglycans. All these features recapitulate human aneurysmal disease, and the latter, involving massive accrual of aggrecan and other proteoglycans, is considered a defining facet of aortic pathology in humans (23, 26, 34). Importantly, YAP expression is decreased in the vascular wall of human aortic aneurysms, suggesting that the mouse model addresses a clinically relevant problem.

A key player in the program of SMC contractile differentiation is the transcription factor myocardin (*Myocd*), which acts together with SRF (*Srf*) to control genes that define mature SMCs, including smooth muscle α-actin (*Acta2*), myosin (*Myh11*), and myosin light chain kinase (*Mylk*) (35–37). Levels of *Myocd* drop in atherosclerosis (38, 39), and altered SMC differentiation, so-called phenotypic modulation, is considered a defining feature of aneurysmal disease (40, 41). Our proteomic and transcriptomic analyses independently support loss of contractile differentiation in i8-YT-KO aortae. The transcriptomic survey indicated reduction in *Myocd*, a finding that was confirmed in independent assays. Loss of contractile differentiation and contractility in i8-YT-KO mice is thus likely a consequence of reduced myocardin-SRF activity. Previous studies have demonstrated that *Myocd* is a downstream target of YAP/TAZ-TEAD signaling (42, 43), acting via a distal enhancer (43). This provides a rational explanation for our finding that SMC differentiation and contractility are reduced in i8-YT-KO mice. A reduction in both systolic and diastolic blood pressures, along with edema formation in the skin, further suggests that reduced arterial contractility and subsequently decreased total peripheral resistance has hemodynamic consequences.

Mutations in target genes of myocardin, including *Myh11*, *Acta2*, and *Mylk*, are known causes of thoracic aortic aneurysms with dissection (TAAD) (2), but most studies show a limited genetic overlap between AAAs and TAAs (44). Among the 3 TAAD-associated genes mentioned above, only MLCK was reduced at the protein level in i8-YT-KO aorta at 2 weeks. No significant reduction in blood pressure or vascular contractility was observed at this time. Thus, we consider it unlikely that the loss in contractile function is the only initiating factor for the development of AAAs in the i8-YT-KO mice. However, it is plausible that the loss of contractile function contributes to the structural dilatation of the thoracic aorta, and potentially, to the progression of AAAs. Differences in the susceptibility of AAAs and TAAs can be partly attributed to the distinct embryological origins of vascular SMCs. Other factors, such as varying hemodynamic forces or the gradual decrease in the elastin-to-collagen ratio along the aortic length, may also contribute to these differences (45).

Recent work identified a role for cGAS/STING-driven proinflammatory signaling after YAP/TAZ deletion in SMCs (5). Evidence was presented that SMC nuclei had reduced integrity, with leakage of double-stranded DNA into the cytoplasm, and that this triggered cGAS/STING signaling and inflammation. The study in question used the *Myh11-CreER^T2^* mouse, which causes a severe visceral pathology when used to delete YAP and TAZ (12, 13). This represents a plausible proinflammatory confounder. Here, using a model that evades intestinal pathology, we demonstrate an increase in the phosphorylated form of the proinflammatory kinase TBK1 in i8-YT-KO aortae. STING was similarly increased at the protein level. We further establish upregulation of a panel of STING–NF-κB–dependent target genes. Our results are thus consistent with increased activation and/or expression of STING and TBK1, which may contribute to inflammation. However, other proinflammatory mechanisms seem likely, and we lack the time resolution to say that STING induction in SMCs precedes aneurysm formation. Importantly, we document intense infiltration of both innate and adaptive immune cell populations into the abdominal aorta, along with accumulation of monocytes and macrophages in the thoracic aorta. This demonstrates considerable innate immune activation and an important role for immune-driven inflammation in this aneurysm model.

The final element of aortic pathology that we examined relates to accumulation of proteoglycans in the aortic media. Using Alcian blue, a dye known to mark acidic polysaccharides, staining was manifest at 2 weeks after induction and before the development of lesions in the thoracic aorta. Although we cannot exclude the involvement of other proteoglycans, our results suggest that accumulation of aggrecan contributes to the increased Alcian blue staining in i8-YT-KO aortae. Our mechanistic studies show increased expression of the chondrogenic transcription factor SOX9, known to directly activate the *Acan* gene (27). Moreover, SOX9 left a significant mark on aortic gene expression in i8-YT-KO mice, and the increase in *Sox9* correlated tightly with the increase in aggrecan, supporting a cause-and-effect relationship. Lineage tracing showed that SOX9 increased in SMCs, and we also found examples of cartilage formation between elastic lamellae. Importantly, *Sox9* induction represented the earliest detectable change in our model, occurring before the proinflammatory transition and phenotypic modulation of SMCs. Because prior studies demonstrated repression of *Sox9* by YAP/TAZ-TEAD (46) and by myocardin (47), we propose that the increased chondrogenic differentiation in the aortic media results from temporally stepwise elimination, or reduction, of important repressive influences on SOX9. It is further notable that matrix molecules, including versican, may act as damage-associated molecular patterns that trigger inflammation via Toll-like receptors (48). In fact, deletion of *Sox9* in cardiomyocytes leads to severely blunted *Il6* production following transaortic constriction (49). It is therefore possible that increased SOX9 expression and the ensuing proteoglycan synthesis contribute to inflammation. The current findings, highlighting a role of SOX9 in aneurysmal disease, align well with the emergent picture of SOX9 as an important culprit in atherosclerosis (50, 51).

To summarize, we present a mouse model for spontaneous AAA formation that bears considerable resemblance to the corresponding human disease. In this model, aneurysms are present in the abdominal aorta 2 weeks after KO induction, and aneurysmal disease later progresses to other vascular territories. Lesions are characterized by proteoglycan accumulation, loss of SMC differentiation, and intense immune cell infiltration. The rapid and consistent disease development is expected to be useful for pharmacological screening and further mechanistic studies. For future studies, it is important to define the role of increased SOX9 expression in aneurysm development following YAP/TAZ deletion, and to target specific immune responses to reduce vascular inflammation in this model.

## Methods

### Human aneurysm samples.

Paired samples from nondilated and dilated tissue biopsies from 15 patients with AAA were utilized. Patient characteristics are described in Supplemental Table 1. Tissue specimens were placed in 700 μL Qiazol and total RNA was isolated using the miRNeasy Micro Kit (Qiagen, 217004) according to the manufacturer’s protocol. RNA concentration and purity were checked using the NanoDrop system (Thermo Fisher Scientific). RNA integrity number (RIN) was assessed using the RNA Screen Tape and the Agilent TapeStation 4200.

### Animals.

Heterozygous *Itga8-CreER^T2^* mice were provided by Lin Gan and Joseph M. Miano (University of Augusta, Georgia, USA) (17), and bred with mice harboring floxed *Yap1* and *Wwtr1* alleles (52) (*Yap1^fl/fl^/Wwtr1^fl/fl^*, strain 030532, The Jackson Laboratory) at Lund University, Sweden to generate *Itga8-Cre ER^T2^*–*Yap1^fl/fl^/Wwtr1^fl/fl^*. For lineage tracing experiments, *Itga8-CreER^T2^*–*Yap1^fl/fl^/Wwtr1^fl/fl^* mice were bred with Rosa mT/mG mice [B6.129(Cg)-*Gt(ROSA)26Sor^tm4(ACTB-tdTomato,-EGFP)Luo^*/J, strain 007676, The Jackson Laboratory] (53). Both male and female mice were used and the data points representing male or female mice are illustrated in the graphs when feasible. To the greatest extent possible, male and female mice were distributed equally in the Ctrl and KO groups. To induce KO, mice were injected intraperitoneally with tamoxifen (1 mg in ethanol/sunflower seed oil, 1:10, as vehicle) for 5 consecutive days at the age of 6 to 8 weeks. Littermates that were negative for *Itga8-CreER^T2^* but harbored floxed alleles for *Yap1* and *Wwtr1* were injected with tamoxifen at the age of 6 to 8 weeks and used as Ctrls.

### Blood pressure.

Blood pressure was measured in awake animals at the indicated time points using the CODA high-throughput system (Kent Scientific Corporation). Mice were trained 3 times prior to the first measurement, to familiarize them with the procedure. Each session consisted of 25 cycles, and only cycles with a tail volume of greater than 10 μL were accepted.

### RNA sequencing and RT-qPCR.

RNA extraction was performed with the RNeasy Mini Kit (Qiagen, 85600), using a QIAcube (Qiagen) workstation as per the manufacturer’s recommendations. RNA concentration and purity were measured in a NanoDrop 2000c (Thermo Fisher Scientific). RNA from Ctrl and i8-YT-KO thoracic aortae was analyzed by Novogene Europe, using 150-bp paired-end sequencing on an Illumina platform. A detailed description of the method can be found in the supplemental material.

RT-qPCR validation was performed using QuantiFast probes (Qiagen, 204256) or QuantiNova probes (Qiagen, 208252), and QuantiTect primer assays (Qiagen, QT02448075, QT00163765, QT00175364, QT00143220, QT00098875, QT00151599, QT01648355, QT00126056, QT01077475, QT01062950, QT00261590, QT00140119, and QT02327626). The StepOnePlus System (Thermo Fisher Scientific) was used to run RT-qPCR reactions. Data were analyzed using the 2^–ΔΔCt^ method.

### Histology.

Arteries were either directly isolated or isolated after perfusion fixation (PBS with 4% formaldehyde and heparin) and further fixed overnight at 4°C. Fixed samples were immersed in PBS with 20% sucrose for 24 hours and subsequently embedded in OCT (Histolab, 45830) and sectioned. Movat Pentachrome Stain Kit (Modified Russell-Movat) from Abcam (ab245884) was used following the manufacturer’s recommendations. For Alcian blue staining, slides were incubated in 3% acetic acid solution for 3 minutes, followed by incubation in Alcian blue solution (Sigma-Aldrich, 1.01647.0500) for 30 minutes at room temperature. The slides were briefly rinsed in 3% acetic acid solution and washed with running tap water and distilled water. Nuclei were counterstained with Nuclear Fast Red solution (Abcam, ab246831) for 5 minutes, followed by running tap water and 2 changes with distilled water. Finally, slides were dehydrated, cleared, and mounted with Pertex (Histolab, 00840-05).

### Immunofluorescence.

Tissue sections were permeabilized with 0.20% Triton X-100 in PBS for 10 minutes, followed by 30 minutes of 3% bovine serum albumin in PBS as blocking solution. Primary antibodies were applied for 90 minutes at room temperature (aggrecan, Rb 76-03; ref. 54) or overnight at 4°C (MLCK, Abcam, ab76092; YAP/TAZ, Cell Signaling Technology, 8418S; Ki67, Abcam, ab15580; TBK1, Cell Signaling Technology, 3504S; SOX9, Abcam, ab185966; CD68, Invitrogen, MCA5709). Donkey or goat anti-rabbit conjugated with Alexa Fluor 555 (Invitrogen, A31572 or A21428) or donkey anti-rabbit conjugated with Alexa Fluor 647 (Invitrogen, A31573) was used as secondary antibody for 1 hour at room temperature. Slides were then incubated for 2 minutes in DAPI (1 μg/mL in PBS) to counterstain for nuclei and mounted in Aqua-PolyMount (Polysciences, 18606-20).

### Immunohistochemistry.

Cryosections were postfixed in acetone (–20°C) and incubated in 3% hydrogen peroxide to inactivate endogenous peroxidase activity. Antigens were then unmasked by treatment with bovine testicular hyaluronidase (Sigma-Aldrich, type I-S, H3506) in acetate buffer (pH 5.0, 30 minutes, room temperature), and nonspecific binding blocked by 2.5% horse serum (Vector Laboratories, S-2012). The sections were incubated with primary antibodies against aggrecan (Rb 76-03, raised against a purified bovine aggrecan G1 fragment and validated in an aggrecan gene–targeting mouse model; ref. 54) and collagen type II (rabbit mAb 1K11, Sigma-Aldrich, ZRB1201) diluted in 2.5% horse serum, overnight at 4°C. Peroxidase polymer-conjugated secondary antibody (ImmPRESS anti-rabbit, Vector Laboratories, MP-7401) was applied for 30 minutes and detected with diaminobenzidine substrate (ImmPACT DAB, Vector Laboratories, SK-4105). Sections were counterstained with hematoxylin QS (Vector Laboratories, H-3404), dehydrated, and mounted in Eukitt mounting medium (Sigma-Aldrich, 03989).

### TUNEL assay.

Click-iT Plus TUNEL Assay for in situ apoptosis detection (Thermo Fisher Scientific, C10618) was performed on prefixed cryosections following the manufacturer’s recommendations. Slides were submerged in DAPI (1 μg/mL in PBS) for 2 minutes to stain nuclei and mounted using Aqua-PolyMount (Polysciences, 18606-20).

### Quantification assays.

Elastin autofluorescence was used to identify individual elastic layers of the aorta. On average, 4 images at different locations along the abdominal aorta were analyzed. For each image, the number of elastic layers was counted at up to 8 fixed positions and the average number of elastic layers/aorta was used for statistics. Media thickness was analyzed at 1 to 4 locations along the abdominal aorta. For each image, media thickness was measured at up to 8 fixed positions/image. The average media thickness/aorta was used for statistics. Lumen circumference was measured at, on average, 3 locations along the thoracic and abdominal aortae and converted to diameter. The average lumen diameter/aorta was used for statistical analysis.

### Godet test.

Edema assessment was done with the Godet test in the hind limbs. The mouse was immobilized, fur was removed locally, and pressure was exerted with a cotton swab for 2 seconds. The swab was lifted, and the time required for the skin to rebound to its initial form was recorded in seconds.

### Ultrasound imaging.

Mice were anesthetized with oxygen and isoflurane (1.5%–2%) throughout the procedure and immobilized on a heated platform (41°C) in the supine position to maintain the body temperature at 37°C. Heart frequency, respiratory rate, and body temperature were continuously monitored. Warmed ultrasound gel was applied to a shaved area of interest and the MX550D probe (40 MHz) was used to obtain B-mode and M-mode images by the imaging system Vevo 3100 (VisualSonics). B- and M-mode images were acquired from the long axis of the heart to obtain accurate measurements of cardiac dimensions. Aortic diameters were measured from the B-mode aortic arch view and scans of abdominal aorta were obtained at the level of right renal arterial branch.

### Electron microscopy.

One 5 mm piece from the distal thoracic aorta per mouse was dissected from i8-YT-KO and Ctrl mice 8 weeks aftyer tamoxifen treatment. Pieces were fixed with 2% paraformaldehyde and 2% glutaraldehyde in 0.1 M Sorensen phosphate buffer. After rinsing in the same buffer, 2% osmium tetroxide was used for postfixation. After dehydration in acetone and embedding in Polybed 812, sections for TEM were cut using a diamond knife and mounted on grids. After staining with 4% uranyl acetate and 1% lead citrate, images were taken acquired a Tecnai BioTwin 120 Kv microscope at ×16,500 and ×87,000 magnification.

### Proteomics.

Aortic tissue was processed using an optimized filter-aided sample preparation, as described previously (55). A detailed description of the method can be found in the supplemental material.

### Western blotting.

Western blot analyses were conducted as previously described (3). Antibodies against the following proteins were used: MLCK (Sigma-Aldrich, M7905; 1:1000), STING (Cell Signaling Technology, 13647S; 1:1000), p-TBK1 (Cell Signaling Technology, 5483; 1:500), TBK1 (Cell Signaling Technology, 3504; 1:500), and SOX9 (Cell Signaling Technology, 82630S; 1:500). HRP-conjugated secondary antibodies were used (Cell Signaling Technology, 7074S and 7076S; 1:10,000) and signals were developed using SuperSignal West Femto Maximum Sensitivity Substrate (Thermo Fisher Scientific, 34095). Images were acquired using an Odyssey Fc instrument and analyzed using Image Studio software (LI-COR Biosciences). Following detection, immunoblots were stripped (Thermo Fisher Scientific, 46430) and reused for detection of total protein levels. See complete unedited blots in the supplemental material.

### Wire myography.

Caudal artery segments (2 mm long) were prepared and mounted using steel wire in myograph chambers (610M and 620M, Danish Myo Technology) containing HEPES-buffered Krebs solution (135.5 mM NaCl, 5.9 mM KCl, 2.5 mM CaCl_2_, 1.2 mM, MgCl_2_, 11.6 mM glucose, and 11.6 mM HEPES, pH 7.4) (56). After heating to 37°C, segments were slowly stretched to a basal tension of 5 mN, and the outer distance between the wires was measured. Following another 25 minutes of equilibration, arteries were activated using 60 mM KCl, cirazoline, and vasopressin. Agonists were purchased from Sigma-Aldrich and dissolved in buffer. Force was continuously monitored using LabChart software (ADInstruments).

### Flow cytometry.

For i8-YT-KO animals, the thoracic and abdominal aortae were divided and analyzed separately. For Ctrl mice, the whole aorta was processed at once. A detailed description of the method can be found in the supplemental material.

### Analyses using GTExPortal data.

Transcriptomic data from human arteries (tibial artery, *n* = 663, aorta, *n* = 432, coronary artery, *n* = 240) were downloaded from https://gtexportal.org/home/ in 2020 (57). *YAP1* (in transcripts per million, TPM) was correlated with all other transcripts in each artery using Pearson’s method. *R* values for panels of MYOCD targets (defined in-house prior to analysis), STING–NF-κB targets (20), and SOX9 targets (28) were subsequently compared to the median *R* value for all transcripts in each artery using Wilcoxon’s 1-sample test in GraphPad Prism. Plotted correlations were tested using Spearman’s method. For stratification, we used the tibial artery data and compared individuals with the highest *YAP1* expression (top 10%) with those with the lowest *YAP1* (bottom 10%) expression. Group-wise comparisons were done for selected genes on the respective panels.

### Statistics.

Analysis and graphing were done in GraphPad Prism 9.5.1. Normality was determined using the Shapiro-Wilks normality test. For normally distributed data, Student’s *t* test was used for comparison of 2 groups. If data did not pass the normality test, the Mann-Whitney test was used. In either case, a 2-tailed *P* value was used. Single-sample data were tested data were tested using Wilcoxon’s signed-rank test. Some comparisons (dose-response curves and blood pressure time-course data) were made using 2-way ANOVA and Bonferroni’s post hoc test. Data are shown as mean ± SEM unless otherwise stated. A *P* value of less than 0.05 was considered statistically significant.

### Study approval.

Human aortic aneurysm samples were retrieved from the Munich Vascular Biobank as described previously (58). The Biobank is approved by the local Hospital Ethics Committee (2799/10, Ethikkommission der Fakultät für Medizin der Technischen Universität München, Munich, Germany), is run in accordance with the Declaration of Helsinki, and all specimens were obtained after informed consent.

All animal experiments were approved by the Malmö/Lund Ethics Committee for Animal Experiments, Lund, Sweden (5.8.18-02990/2020).

### Data availability.

Data are available in public repositories (MassIVE with the identifier MSV000091464; NCBI Gene Expression Omnibus with accession number GSE240233), in Supplemental Data Values, or from the corresponding author upon request.

## Author contributions

MAM was responsible for tamoxifen injections and in vivo experiments. OR did Western blotting and imaging. JAB and TAJ conducted the proteomic experiments. SC performed echocardiography. GJ and AS performed the flow cytometry experiments. FD contributed to early planning and initial experiments. CW collected intestines and documented gross morphology. AA performed immunohistochemical staining for collagen II and aggrecan. LM provided human aneurysm tissue samples and performed qPCR analysis. CR contributed to blood pressure measurements, perfusion fixation, and blood sampling. JH, KS, and SA conceived and planned the work, generated funding, supervised students, and contributed to many of the experiments.

## Supplementary Material

Supplemental data

Supporting data values

## Version 1 08/10/2023

In-Press Preview

## Version 2 09/08/2023

Electronic publication
