# Supplementary material for: Vascular smooth muscle–specific YAP/TAZ deletion triggers aneurysm development in mouse aorta
Source: JCI Insight. 2023 Sep 8;8(17):e170845. doi: 10.1172/jci.insight.170845 (PMC10544211; doi:10.1172/jci.insight.170845)

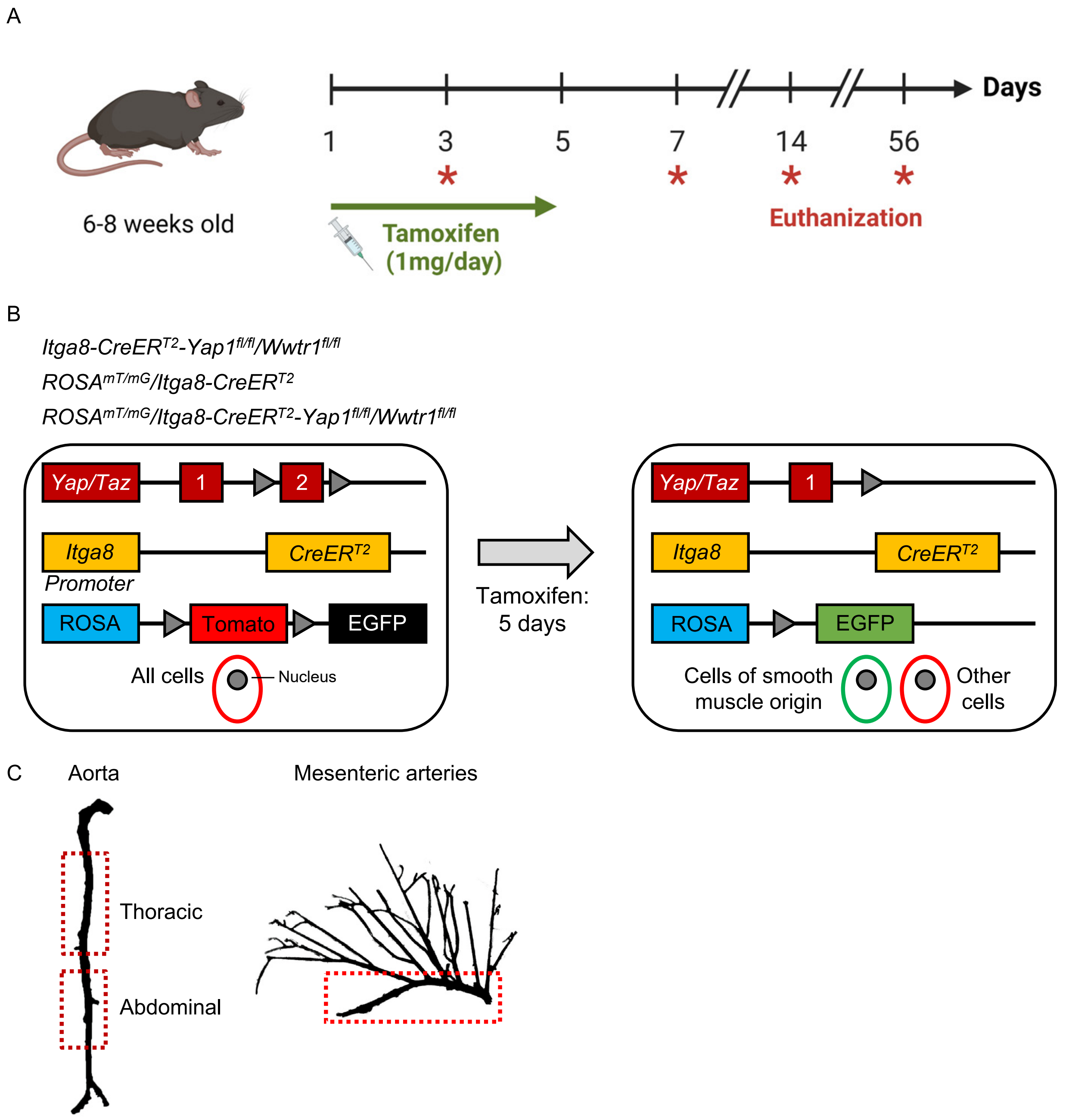

Supplemental Figure 1. An overview of experimental and genetic strategy. Panel A shows timeline representation of knockout induction (tamoxifen injections) and euthanization. Panel B shows a schematic drawing of the *Itga8 Cre-Lox* system. Panel C highlights parts of the aorta and mesenteric arteries that were cryosectioned (red boxes).

Supplemental Fig 1

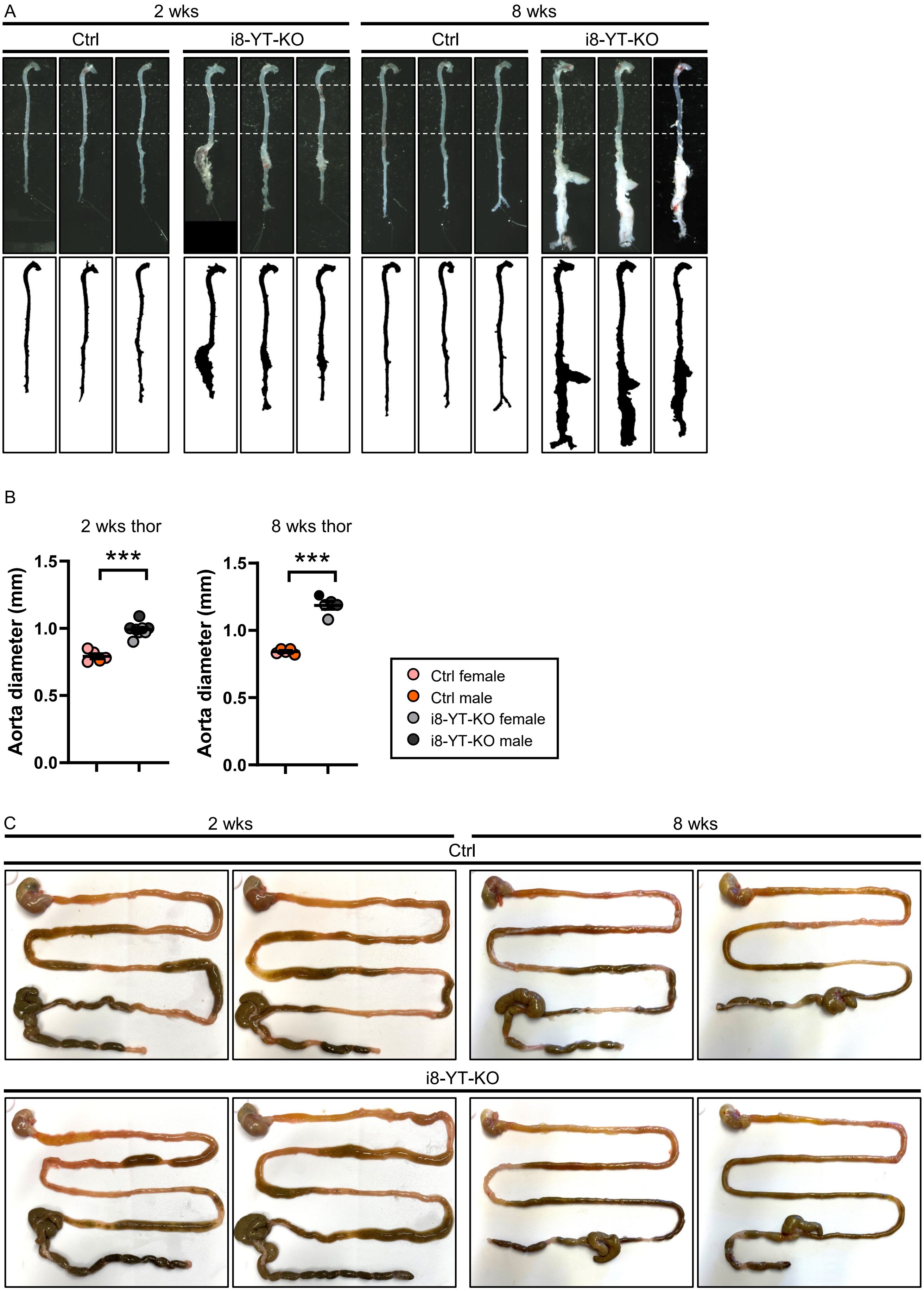

Supplemental Fig 2

Supplemental Figure 2. Aneurysms in abdominal and thoracic aorta arise without gross pathology in the gastrointestinal tract. Panel A shows aortic whole mounts at two and eight weeks post-tamoxifen. Black and white images below are shown for clarity. Quantification of the aortic diameter between the dashed lines in A is shown in panel B (n≥5). Panel C shows a grossly normal gastrointestinal tract in control (Ctrl) and i8-YT-KO mice at two and eight weeks. We previously reported colonic pseudo-obstruction, with enlargement of the caecum and colon following YAP/TAZ knockout using *Myh11-CreER*^{T2}. Only one out of 53 i8-YT-KO mice at eight weeks post-tamoxifen exhibited colonic pathology consistent with obstruction. Student's t-test was used in panel B ***P<0.001.

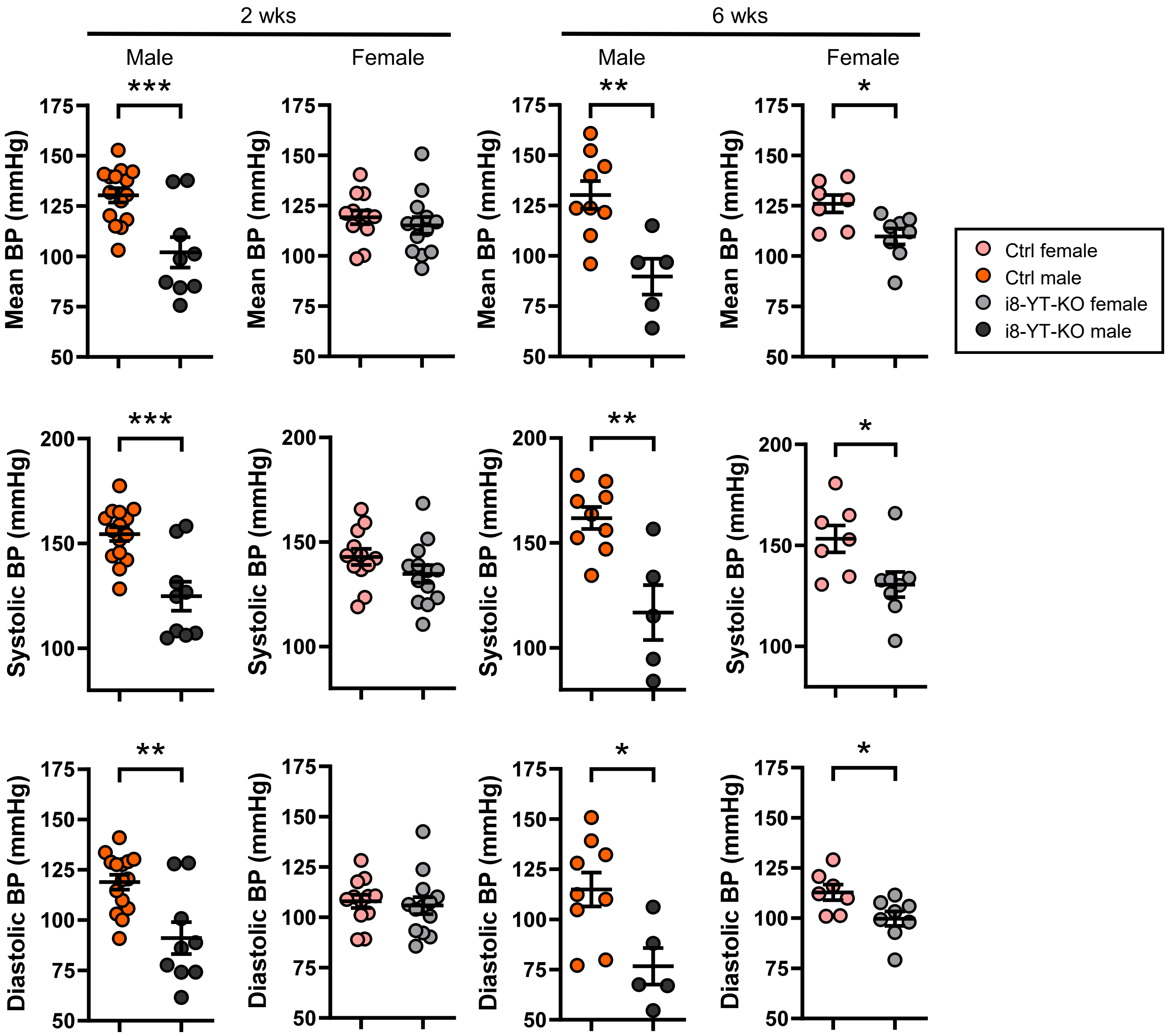

Supplemental Figure 3. Male i8-YT-KO mice display earlier onset of decreased blood pressure than i8-YT-KO female mice. Mean, systolic and diastolic blood pressure (BP) obtained in awake mice by tail-cuff measurement method. Data has been segregated by gender. Student's t-test was used in all graphs; *P<0.05; **P<0.01; ***P<0.001.

Supplemental Fig 3

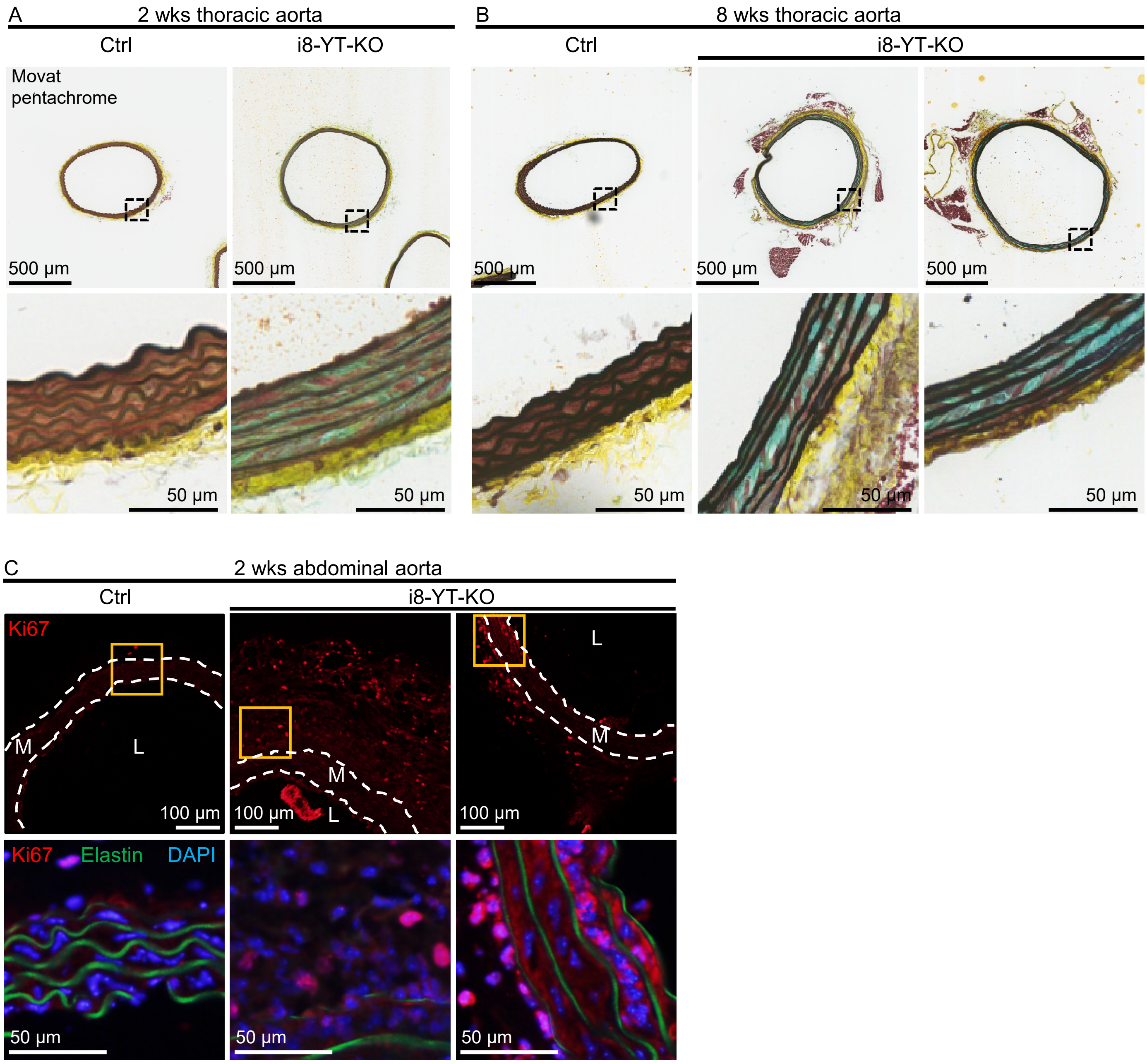

Supplemental Figure 4. Proteoglycan accumulation in the thoracic aorta at two and eight weeks. Aneurysmal lesions were less prevalent in thoracic aorta at two weeks, but medial blue staining (acidic polysaccharides) using Movat pentachrome stain was typically present, and elastic lamellae (black) appeared less wavy (A). Blue staining of the media was further accentuated at eight weeks (B). Ki67 staining, a marker of cell proliferation, was increased in abdominal aorta at two weeks, mainly in the adventitia but occasionally in the media (M) as shown in panel C. DAPI (blue) was used as nuclear stain. L: arterial lumen, and green is autofluorescent elastin.

Supplemental Fig 4

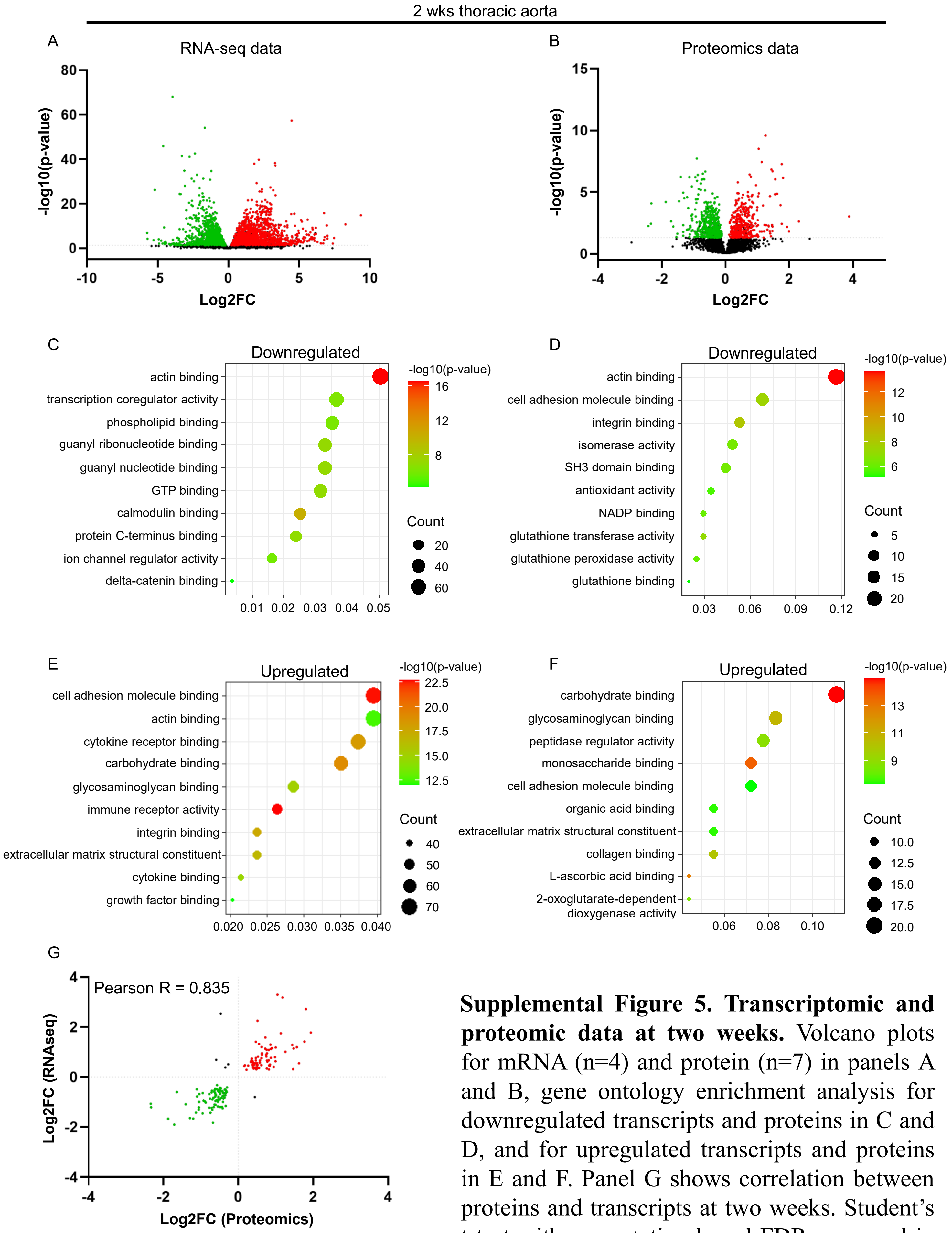

Supplemental Figure 5. Transcriptomic and proteomic data at two weeks. Volcano plots for mRNA (n=4) and protein (n=7) in panels A and B, gene ontology enrichment analysis for downregulated transcripts and proteins in C and D, and for upregulated transcripts and proteins in E and F. Panel G shows correlation between proteins and transcripts at two weeks. Student's t-test with permutation-based FDR was used in panel A-B; Pearson R was used in panel G.

Supplemental Fig 5

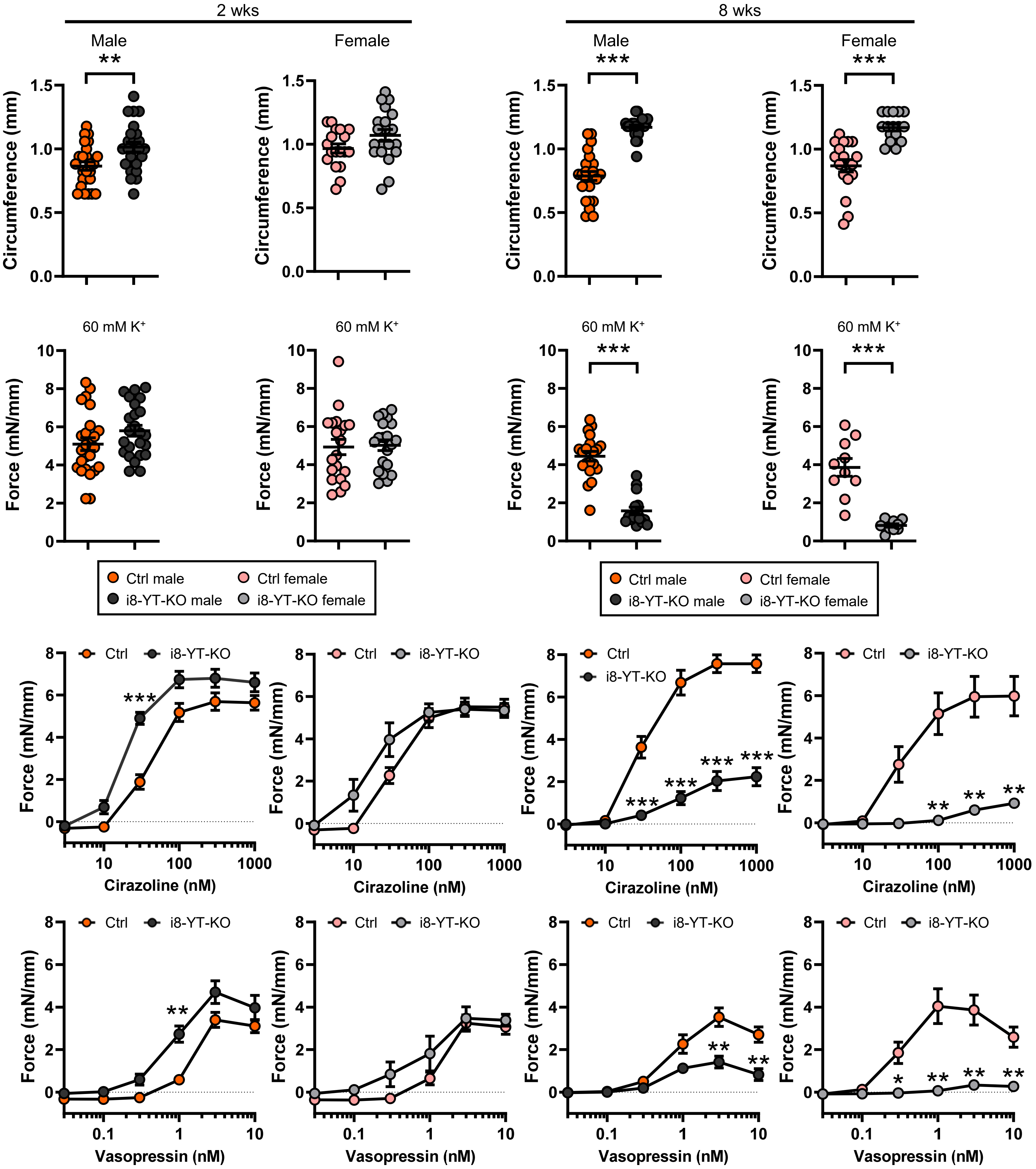

Supplemental Fig 6

Supplemental Figure 6. Male i8-YT-KO mice display earlier onset of increased caudal artery circumference than i8-YT-KO female mice. Tail artery myography data separated by gender. First row: Artery circumference with a pretension force of 5 mN. Only male i8-YT-KO mice show increased circumference two weeks after YAP/TAZ deletion (Ctrl males: 12 mice/25 vessels, i8-YT-KO males: 12 mice/24 vessels, Ctrl females: 9 mice/18 vessels, i8-YT-KO females: 10 mice/20 vessels). Eight weeks after YAP/TAZ deletion both male and female i8-YT-KO mice show increased circumference (Ctrl males: 12 mice/26vessels, i8-YT-KO males: 10 mice/22 vessels, Ctrl females: 9 mice/18 vessels, i8-YT-KO females: 8 mice/16 vessels). Student's t-test was used in the first two graphs and Mann-Whitney was used in the two last graphs. Second row: Contractile force induced by 60 mM K^+ did not differ between male and female mice at two weeks (Ctrl males: 12 mice/25 vessels, i8-YT-KO males: 12 mice/24 vessels, Ctrl females: 9 mice/18 vessels, i8-YT-KO females: 10 mice/20 vessels) or eight weeks after YAP/TAZ deletion (Ctrl males: 9 mice/ 20 vessels, i8-YT-KO males: 7 mice/16 vessels, Ctrl females: 5 mice/10 vessels, i8-YT-KO females: 3 mice/6 vessels). Student's t-test was used in all graphs except the third graph, where Mann-Whitney was used. Third and fourth rows: Male and female i8-YT-KO mice respond similarly to cirazoline and vasopressin, both two weeks (Ctrl males: 6 mice/12 vessels, i8-YT-KO males: 3 mice/6 vessels, Ctrl females: 4 mice/8 vessels, i8-YT-KO females: 3 mice/6 vessels) and eight weeks after YAP/TAZ deletion (Ctrl males: 7 mice/14 vessels, i8-YT-KO males: 5 mice/10 vessels; Ctrl females: 5 mice/10 vessels, i8-YT-KO females: 3 mice/6 vessels). Two-way ANOVA and Bonferroni post-tests were used in all graphs; *$P<0.05$; **$P<0.01$; ***$P<0.001$.

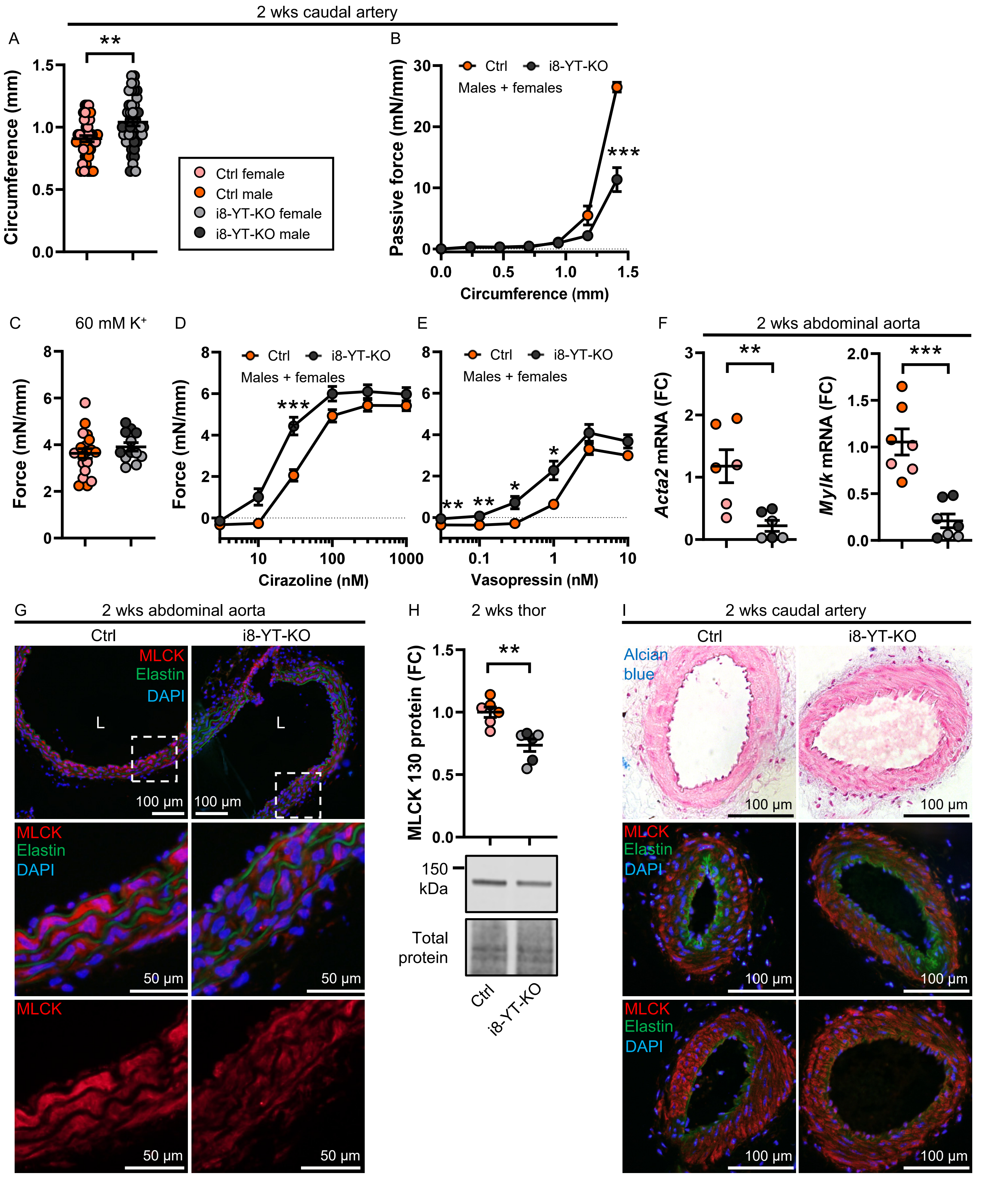

Supplemental Fig 7

Supplemental Figure 7. i8-YT-KO mice show arterial remodeling but maintained or increased contractility at two weeks after YAP/TAZ deletion. Caudal artery segments from 2-week control (Ctrl) and i8-YT-KO mice were mounted in myographs and stretched to a basal tension of 5 mN. The arterial circumference at this tension was calculated from outer wire distance and plotted (A, n≥21 mice and 43 arteries). Full circumference tension relationships confirmed outward remodeling (B). Force in response to 60 mM K^+ (C), cirazoline (D, n≥6 mice and 12 arteries), and vasopressin (E, n≥6 mice and 12 arteries) was unchanged or increased. Panel F shows RT-qPCR for *Acta2* (n=6) and *Mylk* (n=7) in the abdominal aorta at two weeks. Panel G shows immunofluorescence staining for MLCK in abdominal aorta and panel H shows a western blot for MLCK in 2-week thoracic aorta (n=6). In contrast to the clear-cut reduction of MLCK in abdominal aorta at two weeks, no apparent change was seen in caudal artery upon YAP/TAZ deletion (I, bottom). DAPI (blue) was used as nuclear stain. Two-way ANOVA and Bonferroni post-tests were used in panel B, D, E; Student's t-test was used in panel C, F (second graph); *P<0.05; **P<0.01; ***P<0.001.

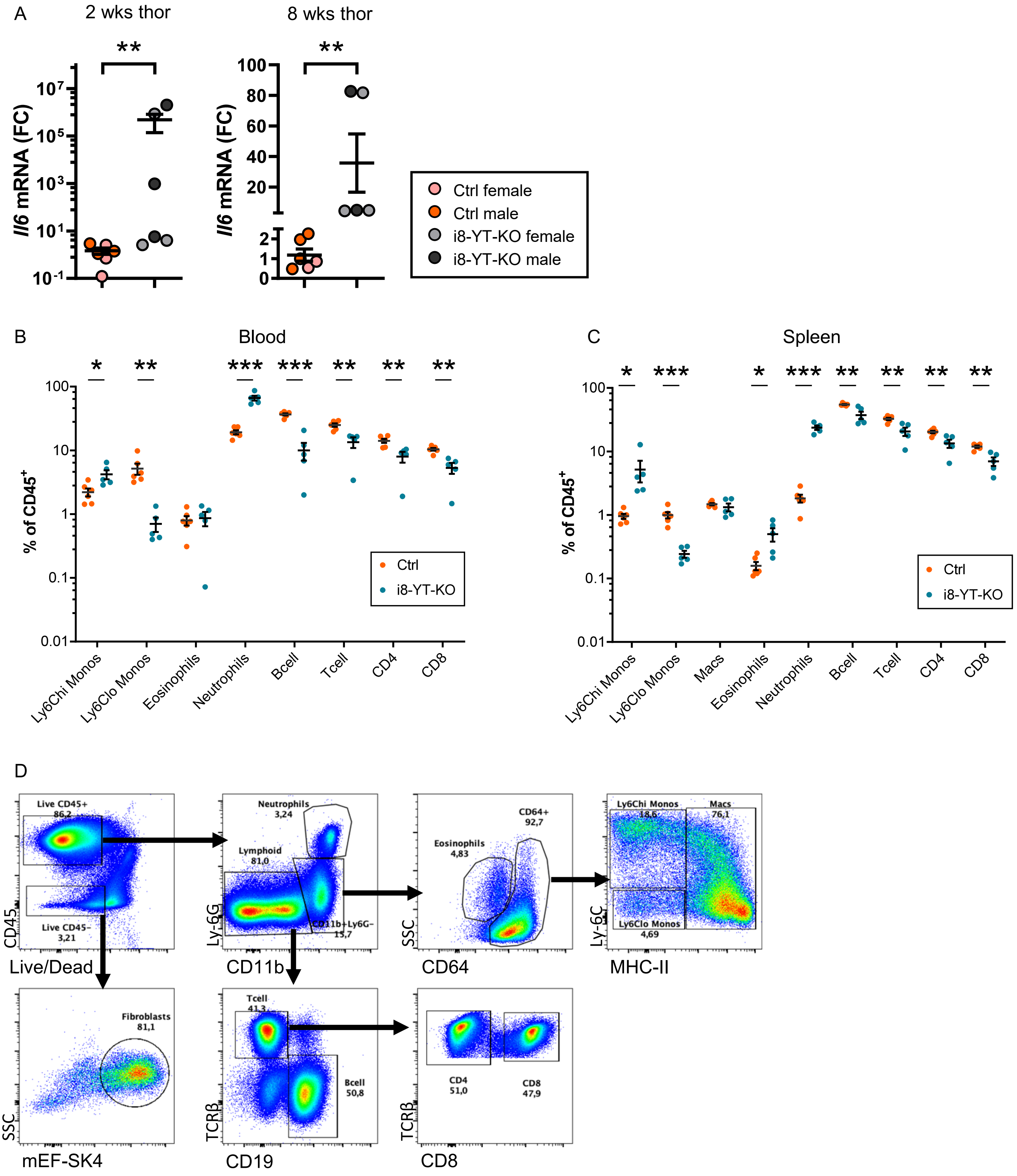

Supplemental Figure 8. Increased neutrophil and inflammatory monocyte percentages in blood and spleen. Panel A shows RT-qPCR for *Il6* in thoracic aorta at two weeks (n≥3) and eight weeks (n≥5) after YAP/TAZ deletion. Panels B and C show flow cytometry data for blood and spleen from Ctrl (orange) and i8-YT-KO mice (blue). Panel D outlines the gating strategy in the flow cytometry experiment. Student's t-test was used in panel A (first graph), B, C; Mann-Whitney was used in panel A (second graph); $^{*}P<0.05$; $^{**}P<0.01$; $^{***}P<0.001$.

Supplemental Fig 8

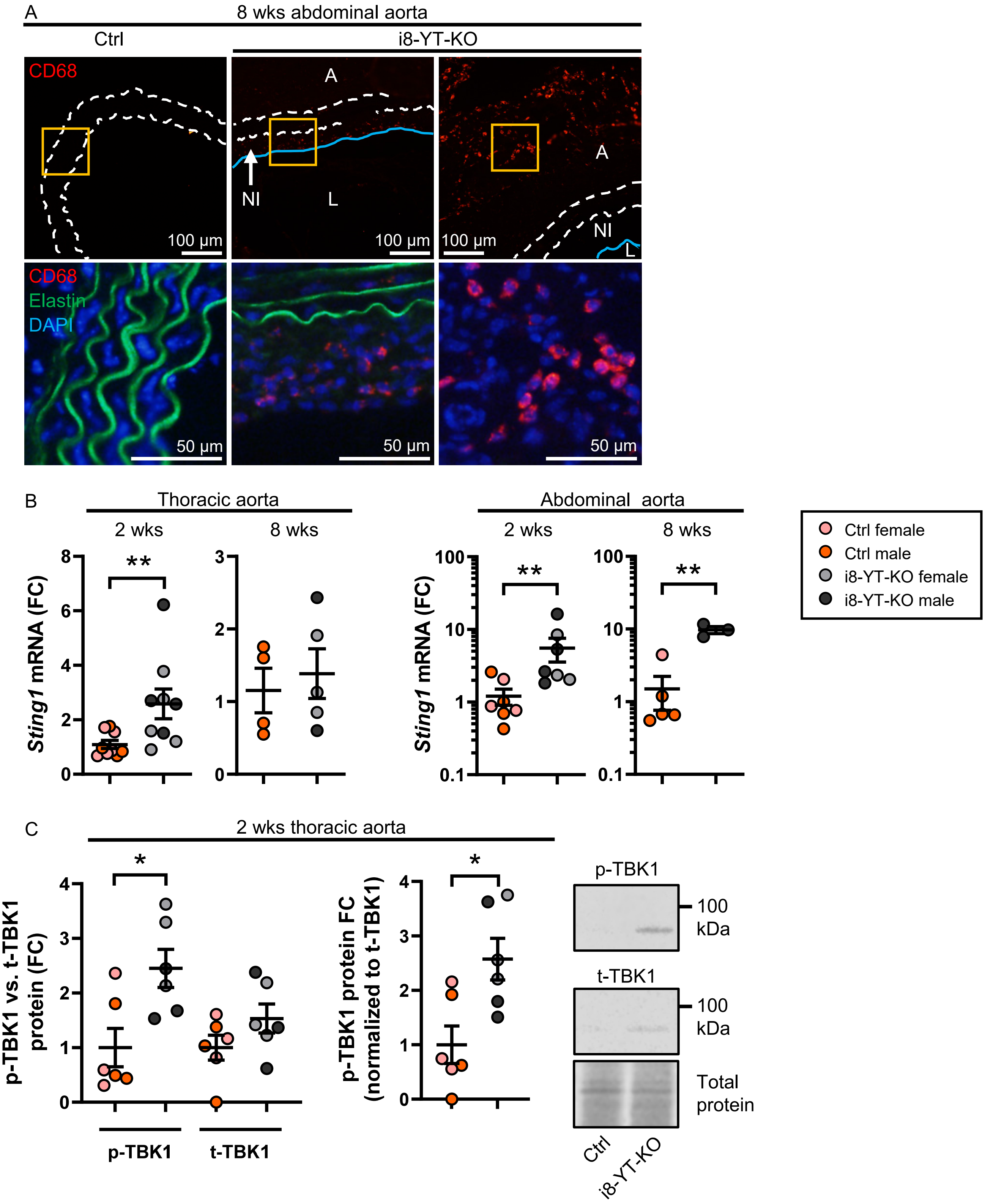

Supplemental Figure 9. Aortae from i8-YT-KO mice display accumulation of macrophages and activation of the STING-pathway. Panel A shows staining for macrophages (CD68, red) in abdominal aorta eight weeks post-tamoxifen. Dashed lines demarcate the media and blue line depicts neointima border. Macrophage staining was seen both in the adventitia and in the neointima. Panel B shows RT-qPCR for *Sting1* in thoracic (n≥4) and abdominal (n≥3) aorta. Panel C shows western blots for total (t)- and phospho (p)-TBK1 in thoracic aorta (n=6). The p-TBK1/t-TBK1 ratio was significantly increased in thoracic aorta as shown in the right graph. DAPI (blue) was used as nuclear stain. A: adventitia, NI: neointima, L: lumen. Student t-test was used in panel B, C (second graph); Mann-Whitney was used in panel C (first graph); *P<0.05; ** P<0.01; ***P<0.001.

Supplemental Fig 9

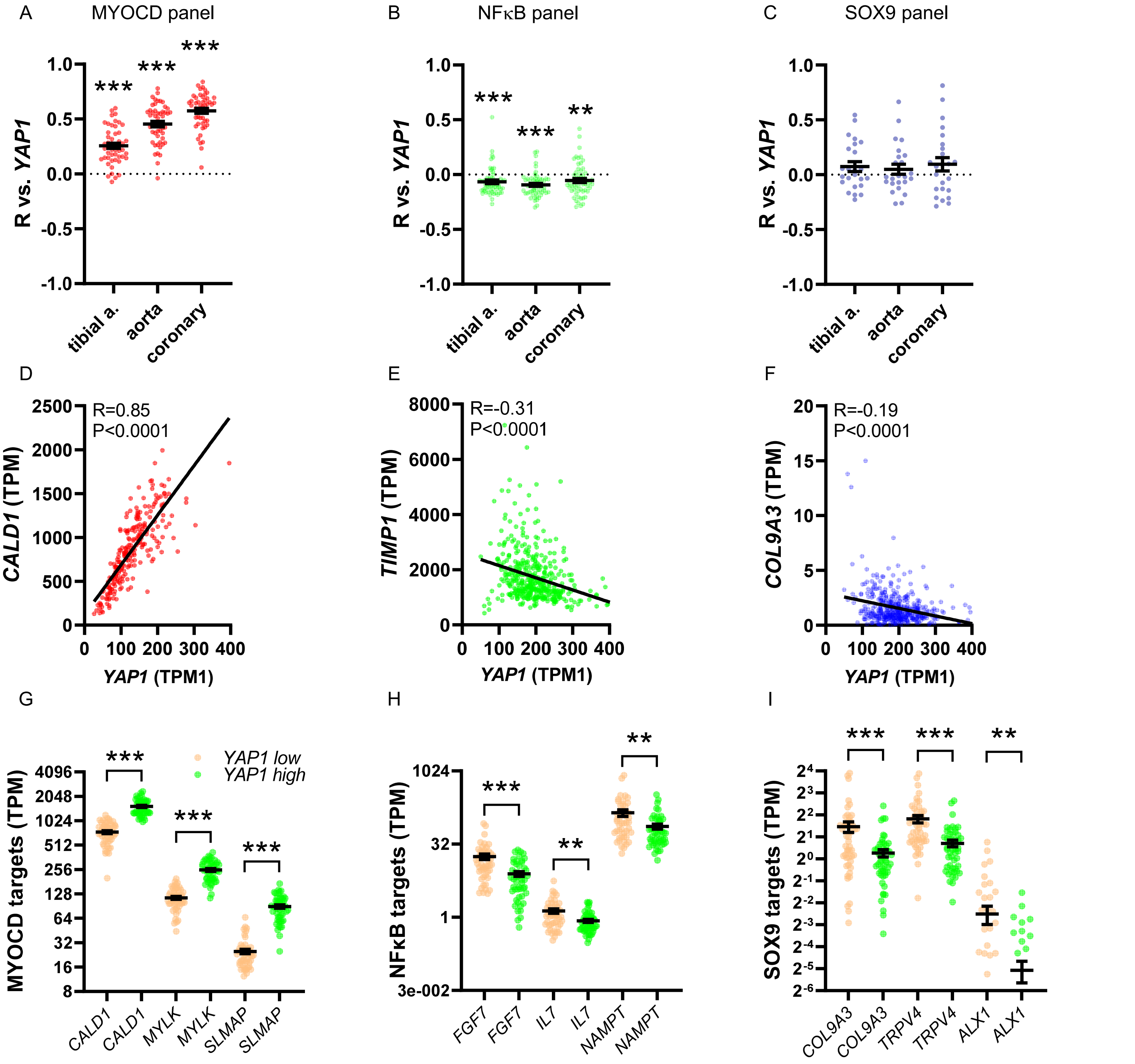

Supplemental Figure 10. *YAP1* associates positively with a panel of myocardin target genes, negatively with a panel of NFκB target genes, whereas no association in either direction is seen for a panel of SOX9 target genes in human arteries. *YAP1* was correlated versus all other transcripts in tibial artery (n=663), aorta (n=432), and coronary artery (n=240), and R-values for the respective groups of target genes were tested versus the median of the entire R-matrix (A-C). Testing was done with a one sample Wilcoxon test. Exemplary correlations are shown in panels D through F. In panels G through I, tibial artery samples were stratified according to level of *YAP1* and the 10% with the lowest level of *YAP1* were compared with the 10% that had the highest level of *YAP1*. Panel G shows examples of myocardin target genes, panel H shows examples of NFκB target genes, and panel I shows examples of SOX9 target genes. Some of the latter did change in the expected direction, even if testing of the entire panel did not show a significant difference for the panel as a whole. All RNA-sequencing data was from GTExPortal.org. Wilcoxon signed-rank test was used in panel A-C; Spearman's correlation was used in panel D-F; Student's t-test was used in panel G (*CALD1, MYLK*); Mann-Whitney was used in panel G (*SLMAP*), H, I; ** P<0.01; ***P<0.001.

Supplemental Fig 10

Patient	Age	Gender	Size (AAD)	Smoking (ever smoked)	Diabetes	Hypertension	Hyperlipidemia	Antiplatelet therapy	Statin therapy
1	75	male	5,5	yes	no	yes	yes	yes	yes
2	67	male	5,6	yes	no	yes	yes	no	yes
3	71	male	6,1	yes	no	yes	yes	yes	yes
4	72	female	5,1	yes	no	yes	yes	no	yes
5	70	male	5,8	no	no	no	yes	no	yes
6	71	male	5,5	no	no	no	yes	yes	yes
7	77	male	7,2	no	no	yes	yes	yes	yes
8	68	male	5,8	yes	no	yes	yes	yes	yes
9	76	female	5,3	no	no	yes	yes	yes	yes
10	73	male	5,9	yes	no	no	yes	yes	yes
11	73	male	5,7	yes	no	yes	yes	yes	yes
12	81	male	6,4	yes	no	yes	yes	yes	yes
13	74	male	5,3	yes	no	yes	yes	yes	yes
14	69	male	6,1	no	no	yes	yes	no	yes
15	75	female	5,5	no	no	yes	yes	yes	yes

AAD: abdominal aorta diameter (cm)

Supplemental Table 1. Basic characteristics of human patients

Supplemental Table 1.

	2 weeks		6 weeks	
	Ctrl	i8-YT-KO	Ctrl	i8-YT-KO
Systolic diameter ascending aorta (mm)	1.31 ± 0.04	1.51 ± 0.04 **	1.14 ± 0.08	1.40 ± 0.06 *
Diastolic diameter ascending aorta (mm)	1.18 ± 0.04	1.30 ± 0.04	1.00 ± 0.02	1.28 ± 0.07 **
Systolic diameter aortic arch (mm)	1.08 ± 0.08	1.14 ± 0.03	1.05 ± 0.05	1.08 ± 0.10
Diastolic diameter aortic arch (mm)	1.00 ± 0.07	1.04 ± 0.04	0.94 ± 0.10	1.10 ± 0.09
Systolic diameter abdominal aorta (mm)	1.03 ± 0.04	1.29 ± 0.05 **	0.93 ± 0.04	1.54 ± 0.21 *
Diastolic diameter abdominal aorta (mm)	0.92 ± 0.04	1.05 ± 0.09	0.74 ± 0.04	1.47 ± 0.22 **

	2 weeks		6 weeks	
	Ctrl	i8-YT-KO	Ctrl	i8-YT-KO
Heart rate (BPM)	508 ± 20	543 ± 24	496 ± 20	500 ± 16
Ejection fraction (%)	74.53 ± 2.70	78.30 ± 1.81	75.36 ± 1.42	84.40 ± 3.44 *
Fractional shortening	42.54 ± 2.43	45.89 ± 1.58	43.26 ± 1.27	53.67 ± 4.51
Cardiac output (ml/min)	15.25 ± 1.39	17.15 ± 1.82	17.78 ± 1.50	14.33 ± 2.29
Stroke volume (µl)	30.26 ± 3.20	31.94 ± 3.81	35.86 ± 2.63	28.93 ± 4.91
End systolic volume (µl)	10.99 ± 2.00	9.35 ± 2.16	11.88 ± 1.25	5.04 ± 1.30 **
End diastolic volume (µl)	40.92 ± 4.71	41.28 ± 5.89	47.74 ± 3.68	34.33 ± 5.89
Systolic diameter (mm)	1.83 ± 0.13	1.73 ± 0.15	1.93 ± 0.09	1.36 ± 0.16 **
Diastolic diameter (mm)	3.17 ± 0.15	3.18 ± 0.19	3.40 ± 0.11	2.92 ± 0.22

Supplemental Table 2. Ultrasound imaging of the aorta and heart in control and i8-YT-KO mice shows aortic enlargement, reduced end systolic volume, and increased ejection fraction at six weeks post-tamoxifen. The top table shows the size of ascending aorta, aortic arch, and abdominal aorta during systole and diastole (n≥4). The bottom table shows the heart function in i8-YT-KO mice at two (n≥5) and six (n=6) weeks after tamoxifen injection. Data for abdominal aorta is duplicated from Figure 1. *P<0.05; **P<0.01; ***P<0.001.

Supplemental Table 2

Actn4	*Ptpn21*	*Zyx*	*Mef2c*	*Pawr*
Npnt	*Dtna*	*Gjc1*	*Efhd1*	*Dusp3*
Igsf9b	*Uba2*	*Tpm1*	*Eml2*	*Myh11*
Ppp1r12b	*Nexn*	*Sacs*	*Fry*	*Mettl24*
Kcnc4	*Ntf3*	*Lmcd1*	*Kctd10*	*Itga7*
Slc20a2	*Dst*	*Rasl12*	*Syde2*	*Acta2*
Rock1	*Myom1*	*Sorbs2*	*Rassf3*	*Lrrfip1*
Cds2	*Pls3*	*Rcan2*	*Fam129a*	*Csrp1*
Lpp	*Ext1*	*Bves*	*Cdkl5*	*Dact3*
Fhl1	*Msrb3*	*Pygo1*	*Nacad*	*Myh10*
Dmd	*Fat1*	*Mark1*		

Supplemental Table 3. List of potential YAP/TAZ target genes. Panel of 53 genes that fulfill all the following criteria (excluding *Yap1*):

1) Included in the top-1000 transcripts that correlate with YAP1 in human aorta,

2) Significantly downregulated (padj<0.001) in *Myh11-CreER*^{T2}-YAP/TAZ KO in Wang et al. (1)

3) Significantly downregulated (padj<0.05) in *Myh11-CreER*^{T2}-YAP/TAZ KO in Daoud et al. (2)

1. Daoud F, Arevalo Martinez M, Holmberg J, Alajbegovic A, Ali N, Rippe C, et al. YAP and TAZ in Vascular Smooth Muscle Confer Protection Against Hypertensive Vasculopathy. *Arterioscler Thromb Vasc Biol.* 2022;42(4):428-43.

2. Wang L, Chennupati R, Jin YJ, Li R, Wang S, Gunther S, et al. YAP/TAZ Are Required to Suppress Osteogenic Differentiation of Vascular Smooth Muscle Cells. *iScience.* 2020;23(12):101860.

Proteomic analysis

In brief, tissue was homogenized in lysis buffer, and protein concentration was quantified using bicinchoninic acid assay (BCA) (Thermo Scientific). After a 5 min heat treatment at 95°C, the sample was diluted in digestion buffer containing 0.5% SDS in 50 mM triethylammonium bicarbonate (TEAB), and reduced and alkylated with 1:50 (v:v) tris(2-carboxyethyl)phosphine (0.5 M, Sigma) and 1:10 (v:v) 2-chloroacetamide (Sigma) for 30 minutes at 37°C. The samples were digested overnight at 37 °C with 1 µg trypsin/LysC mix (Promega) and 0.01% ProteaseMAX (Promega). Peptides were desalted using StageTips containing poly-styrene-divinylbenzene copolymer modified with sulfonic acid groups (SDB-RPS; 3M) material. Following vacuum centrifugation, samples were resuspended in 2% acetonitrile (Sigma) / 0.1% TFA (Sigma). Samples were analyzed in replicates on a Bruker timsTOF Pro mass spectrometer (Bruker Daltonics) operating in DIA PASEF mode with a 1.1 s cycle time and a TIMS ramp time of 100 ms, and the MS scan range was set to 100-1700 m/z. Peptides were separated using an Aurora column with captive spray insert (C18, 1.6 µM particles, 25 cm, 75 µm inner diameter; IonOptics) under controlled temperature at 60°C. A solvent gradient of buffer A (0.1% formic acid) and buffer B (99.9% acetonitrile/0.1% formic acid) was employed over a 42-minutes time period, at a flow rate of 600 nL/min. Protein identification was done using DIA-NN software (version 1.8.0) and the deep learning-based spectra approach to generate a comprehensive library (58) based on the UniProt FASTA database for mouse (UP000000589_10090, Oct. 2022). Protein intensity values were averaged based on the respective technical replicate. Data was log2 transformed and filtered by three valid values in each group. Two-sided Student's t-test with permutation-based FDR (<0.05) and 250 randomizations was used to identify significantly regulated proteins between groups. Correlation analysis and volcano plots were generated in GraphPad Prism (v. 9.5.1). The GO enrichment analysis was performed with R package clusterProfiler using the enrichGO function. MS raw files have been deposited to the public repository MassIVE with the identifier MSV000091464.

Transcriptomic analysis

After cDNA library construction, sequencing, and filtering, reads were mapped to the Mus musculus reference genome (GRCm38/mm10) using Hisat2. Read numbers mapping to each gene were obtained using FeatureCounts and converted to Fragments Per Kilobase of transcript per Million mapped reads (FPKM). Differential gene expression analysis was done using

DESeq2 in R. Enrichment analysis was performed using Gene Ontology (GO) and the Kyoto Encyclopedia of Genes and Genomes (KEGG) using clusterProfiler.

Flow cytometry

Aortic tissue was placed into tubes containing 1 mL digestion mix containing Collagenase I (450 U/ml), Collagenase XI (125 U/ml), DNAase I (60 U/ml), and Hyaluronidase (60 U/ml in Ca^{2+} and Mg^{2+}-free PBS). Tissues were finely minced and incubated for 60 minutes at 37°C with shaking (450 RPM). Single-cell suspensions were obtained by passing through 70 µm cell strainers followed by washing. Blood was isolated using cardiac puncture and subjected to red blood cell lysis using Ammonium Chloride lysis buffer for 3 minutes at room temperature. Spleens were passed through 70 µm cell strainers followed by erythrocyte lysis with Ammonium Chloride lysis buffer for 3 minutes at room temperature.

Single-cell suspensions were stained for viability with LIVE/DEAD™ Fixable Aqua for 10 minutes on ice. Unspecific binding was prevented using CD16/32 for 5 minutes on ice, followed by extracellular antibody staining for 30 minutes on ice. Single-cell suspensions were stained in Mg^{2+} and Ca^{2+}-free PBS supplemented with 0.5% BSA and 2.5 mM EDTA. The following antibodies were used: Ly-6G (1A8), anti-Fibroblast (mEF-SK4), TCRb (H57-597), Ly-6C (HK1.4), CD8 (53-6.7), CD64 (X54-5/7.1), CD45 (30-F11), CD11b (M1/70), CD19 (6D5), MerTK (DS5MMER), MHC-II (M5/114.15.2). Data was collected on a CytoFLEX 3 (Beckman Coulter) and analyzed using FlowJo (Tree Star).

Figure 8I MLCK

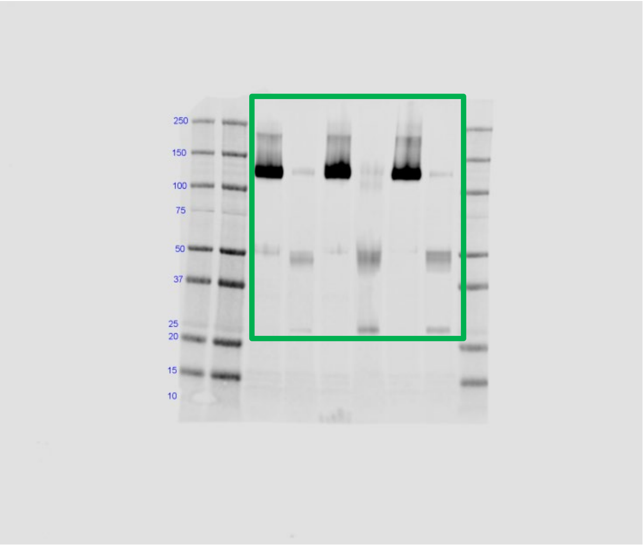

Figure 8K MYH11

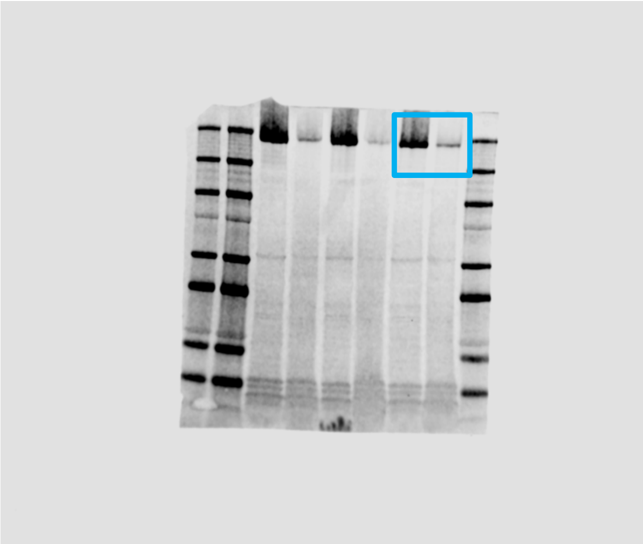

Figure 8I and 8K Total protein

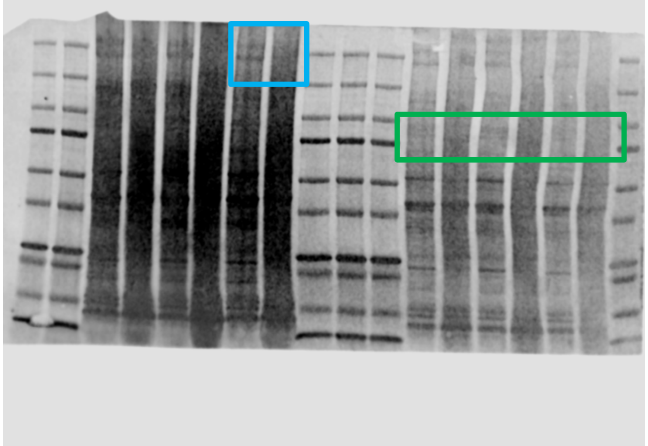

Figure 8J MLCK

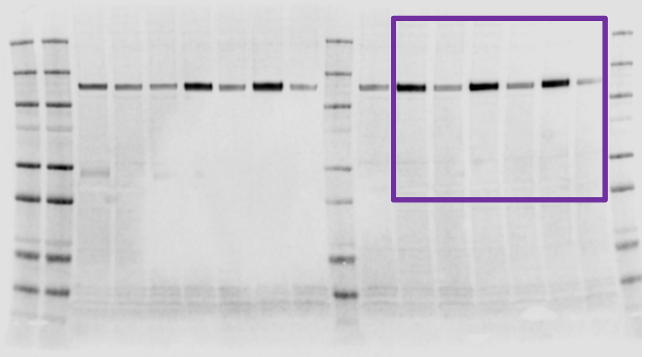

Figure 8J total protein

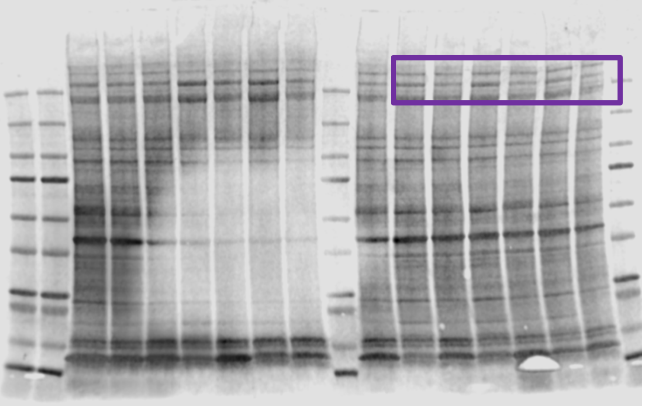

Supplemental Fig 6H Mylk

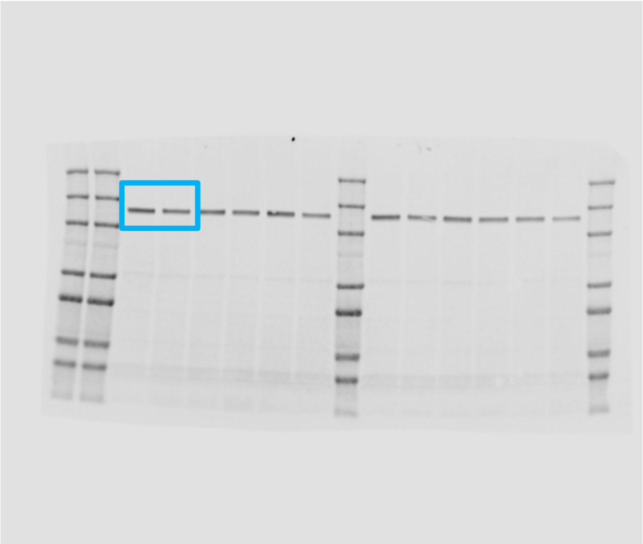

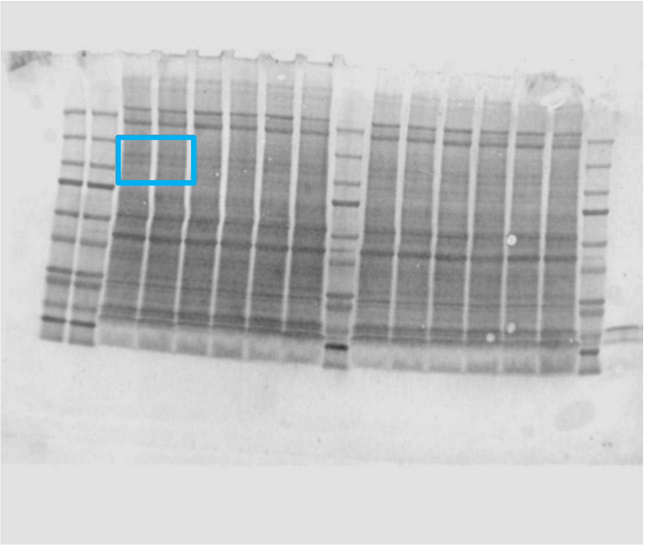

Figure 10D t-STING

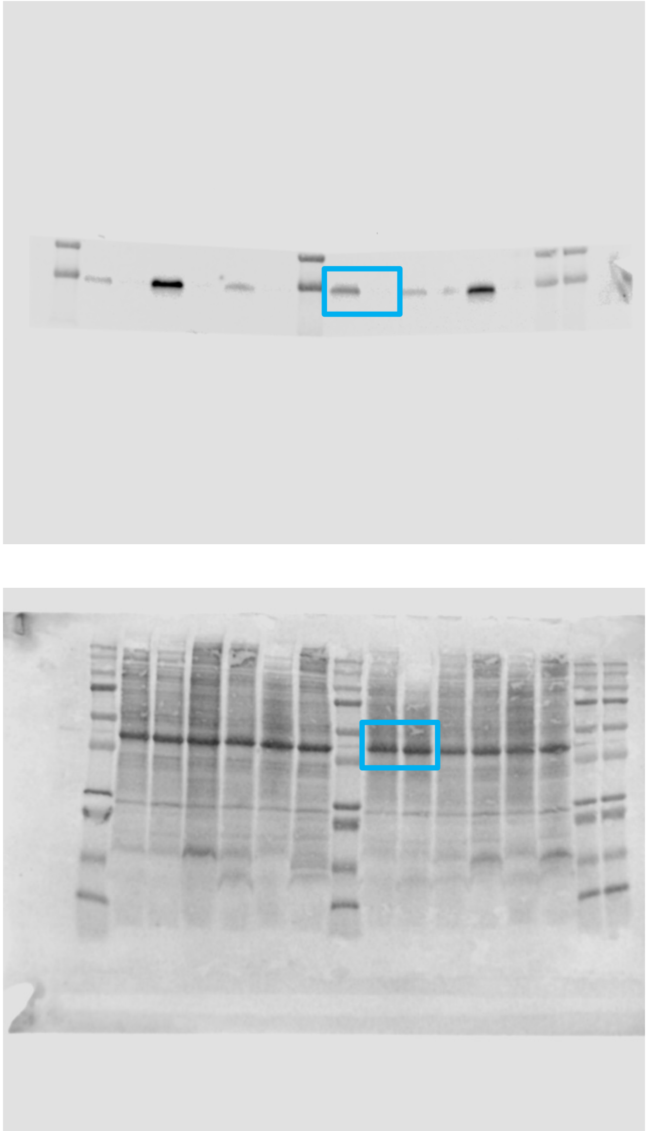

Figure 10D p-TBK1

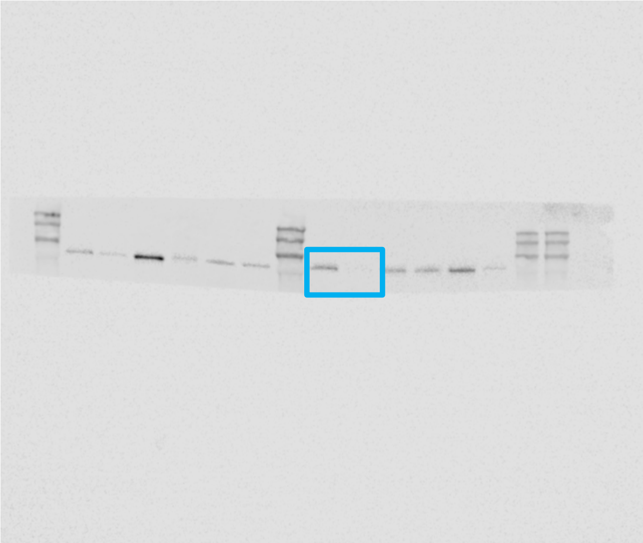

Figure 10D t-TBK1

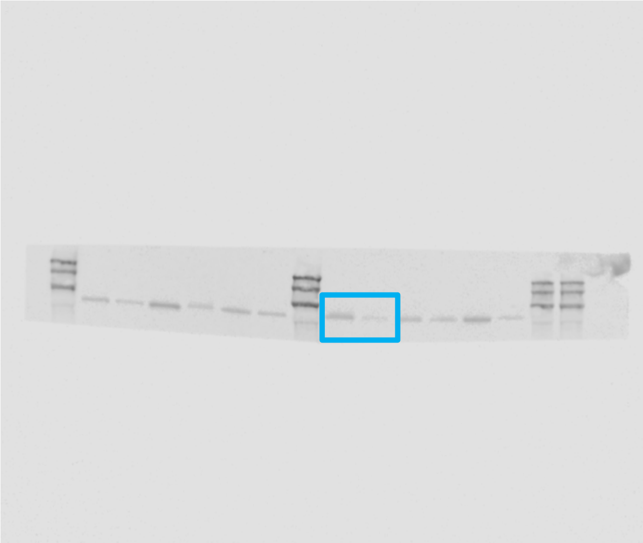

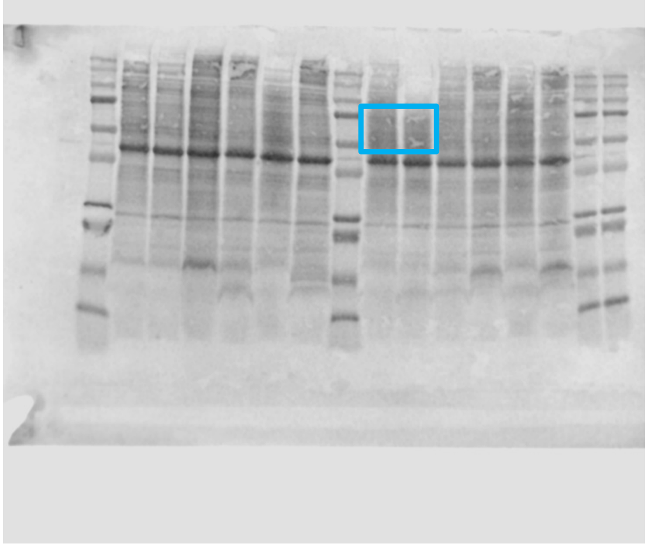

Supplemental Fig 9C p-TBK1

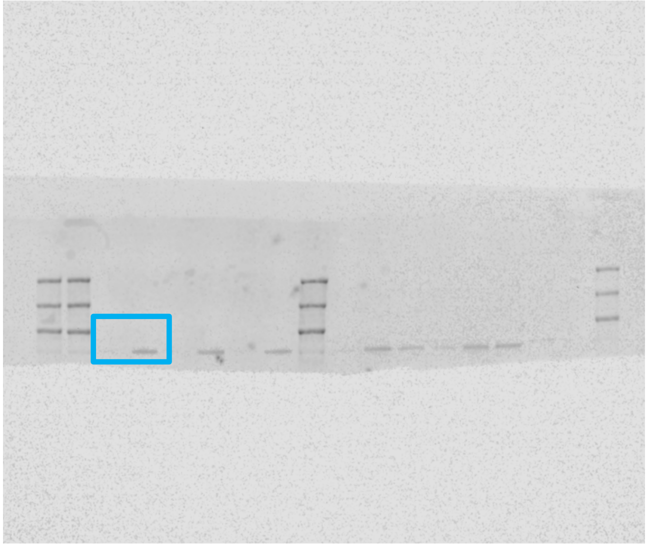

Supplemental Fig 9C t-TBK1

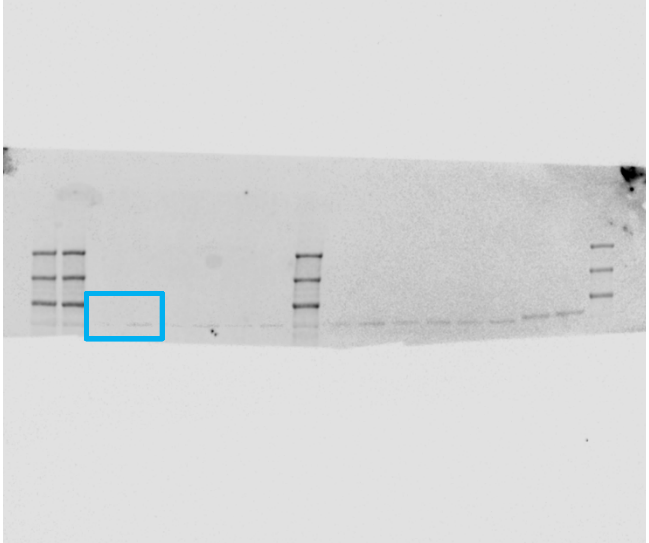

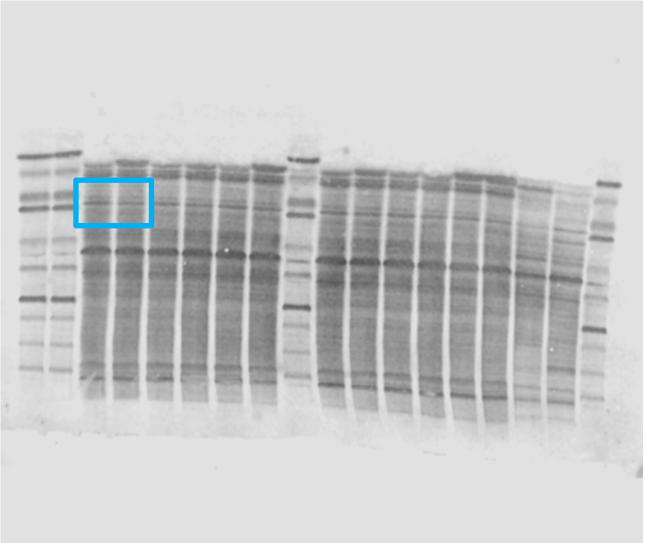

Figure 12A Sox9 (thoracic)

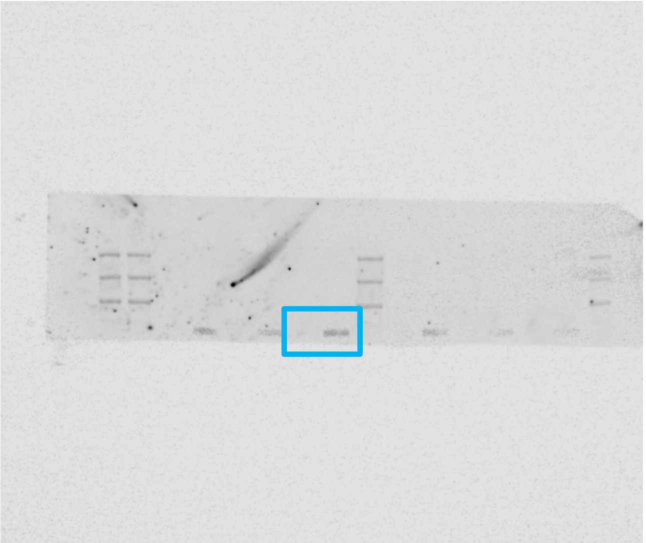

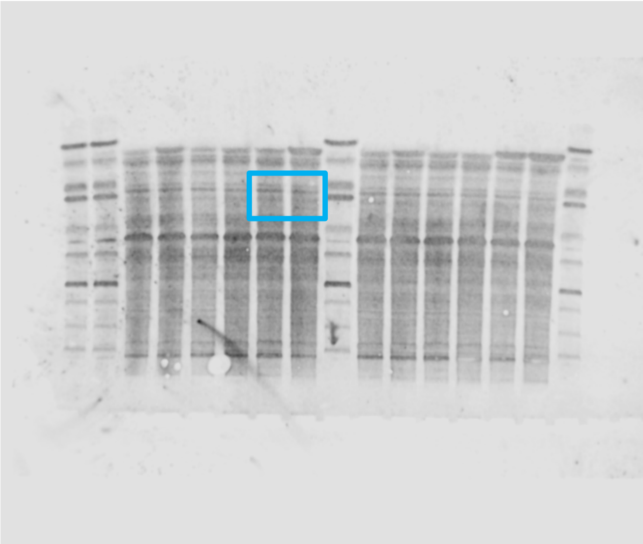

Figure 12A Sox9 (abdominal)

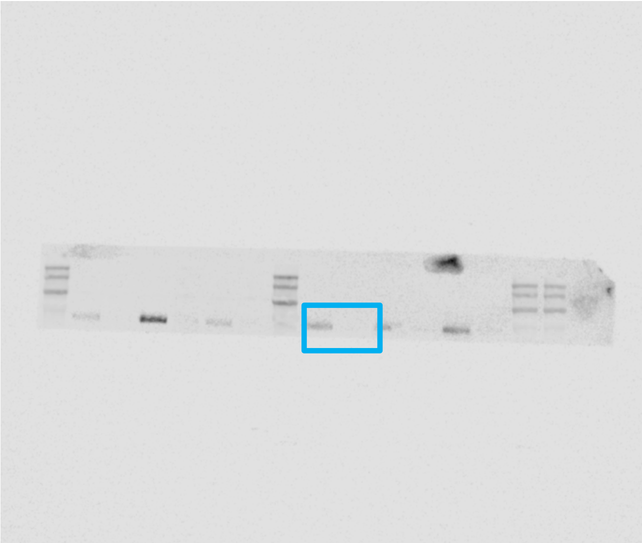

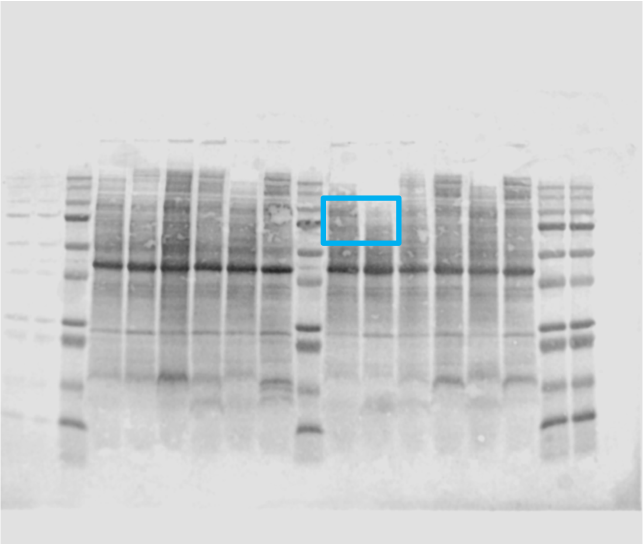

Supplement: Supplemental data [file jciinsight-8-170845-s183.pdf]
